# Multiple factors driving the acquisition efficiency of apple proliferation phytoplasma in *Cacopsylla melanoneura*

**DOI:** 10.1007/s10340-023-01699-1

**Published:** 2023-10-06

**Authors:** Erika Corretto, Massimiliano Trenti, Liliya Štarhová Serbina, James Malcolm Howie, Jessica Dittmer, Christine Kerschbamer, Valentina Candian, Rosemarie Tedeschi, Katrin Janik, Hannes Schuler

**Affiliations:** 1 Faculty of Agricultural, Environmental and Food Sciences, Free University of Bozen-Bolzano, Bozen-Bolzano, Italy; 2 Competence Centre for Plant Health, Free University of Bozen-Bolzano, Bozen-Bolzano, Italy; 3 Laimburg Research Centre, Pfatten-Vadena, Italy; 4 Department of Forest and Soil Sciences, University of Natural Resources and Life Sciences Vienna, BOKU, Vienna, Austria; 5 UMR 1345, Institut Agro, INRAE, IRHS, SFR Quasav, Université d’Angers, Angers, France; 6 Department of Agricultural, Forest and Food Sciences (DISAFA), University of Turin, Grugliasco, Italy

**Keywords:** Psyllids, Insect vectors, Insect-phytoplasma interactions, ‘*Candidatus* Phytoplasma mali’, Cytochrome oxidase I gene (COI), qPCR

## Abstract

Phytoplasmas are bacterial pathogens located in the plant’s phloem that are responsible for several plant diseases and are mainly transmitted by phloem-sucking insects. Apple proliferation (AP) is an economically important disease associated with the presence of ‘*Candidatus* Phytoplasma mali’ which is transmitted by two psyllid species. While *Cacopsylla picta* is a vector in different regions, the vector efficiency of *C. melanoneura* varies between different populations. This species is considered the main AP vector in Northwestern Italy but plays a minor role in Northeastern Italy and other European regions. To investigate whether the psyllid and/or the phytoplasma subtype drive the phytoplasma acquisition in *C. melanoneura*, a phytoplasma acquisition experiment was set up using single mating couples of overwintered individuals from different psyllid populations and phytoplasma subtypes. All analyzed insect populations acquired phytoplasma, but with different efficiencies and concentrations. The main factors driving the acquisition were the phytoplasma subtype and its concentration in the leaves of the infected trees together with the psyllid lineage. The phytoplasma concentration in the psyllids was again influenced by the phytoplasma subtype, the psyllid lineage and the region of origin, whereas the phytoplasma concentration in the leaves and the psyllid haplotype defined with the cytochrome oxidase I gene had only a minor impact on the phytoplasma concentration. This is the first study evaluating the roles of both the psyllid haplotype and the phytoplasma subtype on the acquisition process and highlights the importance of *C. melanoneura* as an additional AP vector.

**Supplementary Information:**

The online version contains supplementary material available at 10.1007/s10340-023-01699-1.

## Key message

- Psyllids are vectors of phytoplasmas associated with several diseases in agricultural production.
- Factors underlying different vector efficiency are currently not known.
- The role of the psyllid and phytoplasma subtype in the acquisition process has been assessed.
- Both the phytoplasma subtype and psyllid genetics drive the acquisition process.
- This work increases the understanding of the interactions between insect vectors and phytoplasmas.

## Introduction

Phytoplasmas are cell wall-lacking bacteria (Mollicutes) that have adapted to live in the phloem of a wide range of plants (crops, weeds, trees, wild plants) and insects (Rao et al. 2018; Bertaccini et al. 2019). The insects that act as vectors belong to the families Cicadellidae (leafhoppers), Psyllidae (psyllids) and to the superfamily Fulgoroidea (planthoppers) (Bertaccini et al. 2019). There are currently 49 ‘*Candidatus* Phytoplasma’ species (Bertaccini et al. 2022), which secrete several effector proteins that mediate the interaction with the hosts and are involved in the manipulation of the host plant’s metabolism (Kube et al. 2011; Firrao et al. 2013; Luge et al. 2014; Janik et al. 2017; Namba 2019; Huang et al. 2022). In plants, the metabolic changes induced by the phytoplasma presence cause severe diseases and can lead to decline and death (Rao et al. 2018). The epidemiology of different phytoplasmas depends greatly on the acquisition efficiency of its insect vector, and their transmission is influenced by the insect’s host range and dispersal ability (Alma et al. 2019). Nevertheless, many factors and molecular mechanisms contributing to a successful phytoplasma acquisition and transmission are still unclear due to the complexity of the insect–phytoplasma–plant interaction, and the difficulties related to the reproducibility of such systems in laboratories or greenhouses.

Fruit crops are among the most affected plants (Fiore et al. 2018). In particular, apple proliferation (AP) disease is associated with the presence of ‘*Candidatus* Phytoplasma mali’ (Seemüller and Schneider 2004). The main symptoms consist in enlarged stipules, the formation of branches with the characteristic witches’ broom appearance, early leaf reddening, pre-harvest chlorosis and the production of small, taste- and colorless fruits (Seemüller et al. 2011). The uprooting of symptomatic apple trees together with pesticide treatments to control the insect vectors are the only recommended measures currently available to counteract the spreading of AP phytoplasma.

‘*Candidatus* Phytoplasma mali’ belongs to the 16SrX apple proliferation group (Seemüller and Schneider 2004). It has a linear genome of ca. 600 kb with a low GC content and several repetitive regions (Kube et al. 2008). Based on the sequence analysis of a putative nitroreductase, different subtypes of ‘*Ca.* P. mali’ were defined as AT, AT1, AT2 and AP15 (Jarausch et al. 2000). Their distribution varies within the Northern Italian regions: subtype AT1 is prevalent in Valle d’Aosta, Piemonte and Lombardia (Northwest, NW) (Casati et al. 2010), whereas subtypes AT2 and AP15 are prevalent in Trentino - Alto Adige and Friuli - Venezia - Giulia (Northeast, NE), respectively (Cainelli et al. 2004; Martini et al. 2008). Since ‘*Ca.* P. mali’ resides in the plant’s phloem, it is subjected to seasonal colonization dynamics. During winter, it is located in the roots, while during spring, it migrates back to the aerial parts along with the phloem content (Pedrazzoli et al. 2008).

*Cacopsylla melanoneura* Förster and *C. picta* Förster (Hemiptera: Psylloidea: Psyllidae) are considered the main insect vectors of ‘*Ca.* P. mali’ (Frisinghelli et al. 2000; Tedeschi and Alma 2004; Jarausch et al. 2019). They are univoltine phloem-feeding hemipterans, which overwinter as adults on conifers and migrate for reproduction to apple trees at the beginning of spring (Hodkinson 2009; Oppedisano et al. 2019; Candian et al. 2020). Both vectors acquire phytoplasma by feeding on the phloem of infected plants. The acquisition and transmission process consists of three phases: acquisition (AAP), latency (LP) and inoculation (IAP) (Bosco and D’Amelio 2009; Alma et al. 2019). The effects of ‘*Ca.* P. mali’ on the psyllid behavior and fitness have been best studied in *C. picta*, where adults are not affected by the presence of ‘*Ca*. P. mali’, whereas immatures developing on infected trees are smaller and have a higher mortality rate (Mayer et al. 2008a, b). Interestingly, overwintered AP-positive *C. picta* tend to deposit a higher number of eggs on healthy trees (Mayer et al. 2011). In contrast, ‘*Ca.* P. mali’ does not affect *C. melanoneura*’s development and survival, but causes a lower number of laid eggs on infected plants and a lower percentage of egg hatching rate (Malagnini et al. 2010). A transcriptomic study comparing infected and non-infected adults of *C. melanoneura* showed that the presence of ‘*Ca.* P. mali’ influences several metabolic pathways (i.e., circadian clock, immune and nervous system, carbohydrate metabolism), highlighting its broad impact on the psyllid’s physiology (Weil et al. 2020).

AP is widespread across many European countries, and severe AP outbreaks have been recorded especially in intensive apple-growing regions (Janik et al. 2020). The natural infection rate and transmission capacity of *C. melanoneura* and *C. picta* are heterogeneous among the European regions. Wherever *C. picta* is present, it is considered the main vector, whereas *C. melanoneura* plays an important role only in the regions where *C. picta* is absent (Mattedi et al. 2008). Italy constitutes an interesting area: *C. picta* is exclusively present in the NE, where it is considered the main vector (Oppedisano et al. 2019). On the other hand, *C. melanoneura* is present both in NE and NW, but it is considered a main vector only in the NW, such as Piemonte and Valle d’Aosta (Tedeschi and Alma 2004; Tedeschi et al. 2012; Fischnaller et al. 2017; Candian et al. 2020). This has been demonstrated in transmission studies where *C. melanoneura* collected in NE Italy was less efficient both in acquisition and transmission of ‘*Ca.* P. mali’ compared to *C. picta* (Oppedisano et al. 2019). Therefore, it has been speculated that the variation in *C. melanoneura* vector competence might be linked to genetic differences among distinct populations (Tedeschi and Nardi 2010; Malagnini et al. 2013). Additionally, different populations of *C. melanoneura* seem to be associated with different subtypes of ‘*Ca.* P. mali,’ due to the heterogeneous distribution of its subtypes mentioned above. Thus, in NW Italy, *C. melanoneura* is associated with subtype AT1 (Casati et al. 2010); in contrast, in NE Italy the prevalent subtype is AT2, although AT1 is present in some parts of the region (Cainelli et al. 2004; Baric et al. 2011).

The existence of different vector species and phytoplasma strains in Northern Italy offers the potential to investigate the factors driving vector competence in this pathosystem. This study aims (i) to assess the phytoplasma acquisition efficiency of different *C. melanoneura* populations from NW and NE Italy and (ii) to identify factors driving phytoplasma acquisition and underlying the discrepancy in acquisition efficiency. For this purpose, a full-factorial phytoplasma acquisition experiment was performed starting with single mating couples of *C. melanoneura* from several populations that were reared on trees infected with different ‘*Ca.* P. mali’ subtypes.

## Materials and methods

### Collection and identification of *Cacopsylla melanoneura*

**Fig. 1:**
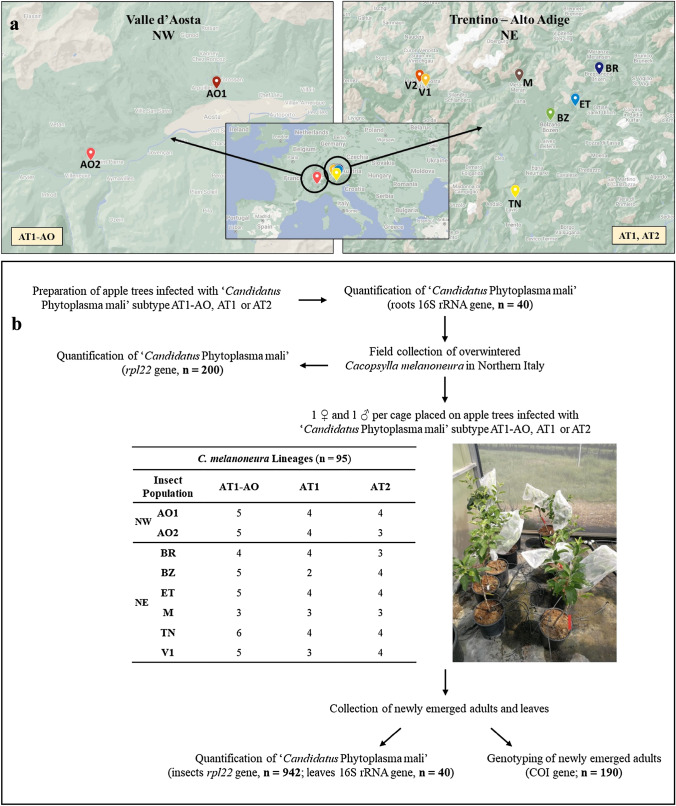
**a** Sampling locations in the region of Valle d’Aosta (Northwest Italy, NW): Aosta–AO1; Saint-Pierre–AO2 and in the region of Trentino—Alto Adige (Northeast Italy, NE): Brixen—BR; Bozen—BZ; Barbian (Eisacktal)—ET; Dorf Tirol (Meran)—M; San Michele all’Adige (Trentino)—TN; Schluderns (Vinschgau)—V1 and Mals (Vinschgau)—V2. The yellow boxes indicate the prevalent ‘*Candidatus* Phytoplasma mali’ subtypes in the corresponding regions: AT1–AO in NW; AT1 and AT2 in NE. **b** Scheme of the phytoplasma acquisition experiment. See materials and methods for details

*Cacopsylla melanoneura* individuals were collected in apple orchards using the beating tray method in the following locations in Northern Italy: two in Valle d’Aosta (Aosta–AO1; Saint-Pierre–AO2) representing the Northwestern area (NW) where *C. melanoneura* acts as vector of AP phytoplasma; one in Trentino (San Michele all’Adige—TN) and six in Alto Adige (Brixen—BR; Bozen—BZ; Barbian, Eisacktal—ET; Dorf Tirol, Meran—M; Schluderns, Vinschgau—V1; Mals, Vinschgau—V2) representing the Northeastern area (NE) where the role of *C. melanoneura* as AP phytoplasma vector is unclear (Fig. 1a). The geographical coordinates of all sampling sites are listed in Table S1. The sampling was performed in March 2021, when the overwintered adults of *C. melanoneura* remigrated from conifers back to the apple trees before mating and oviposition.

The insects were isolated in glass collection tubes for taxonomic identification with a stereomicroscope using dichotomous keys (Ossiannilsson 1992). Males and females were separated and sorted into mating couples for the phytoplasma acquisition experiment. The remaining insects were quickly frozen and stored in absolute ethanol at -20 °C for further analyses. A subset of overwintered *C. melanoneura* individuals was used to determine the phytoplasma infection rate of the natural populations.

### Plant material

Golden Delicious apple trees were grafted using branches from phytoplasma infected trees from orchards in Aosta (Northwest Italy, NW), Brixen and Fragsburg (Northeast Italy, NE). The branches from Aosta carried the subtype AT1 (hereafter named AT1–AO), whereas the material from Brixen and Fragsburg carried AT1 and AT2, respectively. Trees 1–13 and 28–30 were grafted in autumn 2020, whereas the other trees were already prepared in 2016. A PCR analysis with the primers AP10–AP13 (Jarausch et al. 2000) was performed to confirm the subtype of ‘*Ca.* P. mali’ present in the infected material collected in the field. PCR conditions are described in the supplementary information (SI).

### DNA extraction and quantification of phytoplasma in roots and leaves

To confirm the success of the grafting and infection, the presence of ‘*Ca.* P. mali’ was quantified via qPCR analysis of the root phloem before the start of the acquisition experiment. Pieces of roots were collected from different parts of the root system in a non-destructive manner in order not to damage the trees. Subsequently, the phloem was extracted and pooled to obtain around 100 mg of starting material for the DNA extraction. Only trees with phytoplasma positive roots were used for the acquisition experiment (*n* = 40). Additionally, the phytoplasma concentration was measured in the leaves when the newly emerged adults of *C.* *melanoneura* of the acquisition experiment were collected (see details below) (Fig. 1a). DNA of roots and leaves was extracted with the DNeasy Plant Kit (Qiagen) following the manufacturer instructions.

‘*Candidatus* Phytoplasma mali’ was quantified with duplex TaqMan qPCR targeting the phytoplasma 16S rRNA gene and the chloroplast gene for the leucine tRNA as internal positive control as described in Baric and Dalla-Via (2004). PCR conditions are reported in the SI.

### Setup of the phytoplasma acquisition experiment

Overwintered adults, one male and one female of *C. melanoneura,* were placed in single net cages to form mating couples (*n* = 95). The cages were put on single branches of Golden Delicious apple trees infected with ‘*Ca.* P. mali’ subtypes AT1–AO and AT1 or AT2. A total of 24 combinations of *C. melanoneura* populations and phytoplasma subtypes were established, with 3–6 replicate mating couples per combination (Fig. 1b). The plants were kept in the greenhouse covered by an insect-proof net under the following conditions: 26°C during the day, 21°C during the night and 50% relative humidity. Supplemental light was applied using lamps to maintain a 16-h light and 8-h dark cycle. The newly emerged full siblings were allowed to develop completely on AP-infected plants and adults were collected after approximately 10 days. qPCR was used to quantify the phytoplasma concentration in 10 full siblings derived from each mating couple (hereafter referred to as lineage), resulting in a total of 942 individuals analyzed. Additionally, leaves from each tree were collected to verify the infection status of the plant on which the offspring developed.

### DNA extraction and quantification of phytoplasma in insects

DNA extraction from single insects was performed with the DNeasy 96 Blood and Tissue Kit (Qiagen). First, a SYBR Green real time PCR was performed using primers targeting the insect single copy gene wingless (*wg*), qPSY-WG-F and qPSY-WG-R, to verify the quality of the extracted DNA (Brower and DeSalle 1998). Subsequently, the concentration of ‘*Ca.* P. mali’ was measured via SYBR Green qPCR with the primers targeting the *rpl22* gene, rpAP15f-mod and rpAP15r3 (Monti et al. 2013). PCR conditions are reported in the SI.

All insects were tested individually, and each sample was tested in triplicates. Three non-template controls (NTC), containing water instead of the genomic template DNA, were additionally analyzed in every qPCR run. As standards, a four-point tenfold dilution series of the plasmids pJET1.2-wg containing the subcloned *wg* PCR amplicon and pJET1.2-rpl22 with the ‘*Ca.* P. mali’ *rpl22* PCR amplicon were used (Mittelberger et al. 2017). The standards were present in every qPCR run, and the resulting four-point standard curve was used for the quantification of the phytoplasma concentration as phytoplasma copies per insect. Samples were considered positive with a mean *C*_*q*_ value inside the four-point standard curve and if they had a melting curve comparable with the one of the standards. Samples having an additional small peak in their melting curve (e.g., due to primer-dimer formation in samples with very low template concentrations) were considered positive but non-quantifiable (nq). Data analysis was performed using the CFX MANAGER software (Bio-Rad), and only runs with 95–105% efficiency and a determination coefficient of (*R*_2_ ≥ 0.99) were considered.

### Genotyping of the *Cacopsylla melanoneura* lineages from the acquisition experiment

A genetic characterization of *C. melanoneura* populations used in the phytoplasma acquisition experiment was performed as follows: two newly emerged adults per lineage (*n* = 190) were genotyped amplifying the mitochondrial cytochrome oxidase I gene (COI) (Oettl and Schlink 2015). PCR conditions are reported in the SI.

PCR products were sent for sequencing to Eurofins Genomics (Ebersberg, Germany) and then, analyzed by BLAST search (Altschul et al. 1990) using the GenBank database to find the closest described haplotype. The sequences were aligned with MUSCLE (Edgar 2004), and the haplotypes described by Oettl and Schlick (2015) were used as references for the identification of SNPs. Each SNP was confirmed to be present in both individuals. The sequences of the novel haplotypes identified in this study were deposited in the NCBI GenBank database with the accession numbers OQ304120 to OQ304128.

### Statistical analysis of the factors influencing phytoplasma acquisition

We fitted a series of Generalized Linear Mixed Effects Models (GLME) to our acquisition data to test which factors are driving the acquisition of ‘*Ca.* P. mali’ in newly emerged *C. melanoneura* from the acquisition experiment. The considered factors were (i) the psyllid’s region of origin (Valle d’Aosta, Northwest Italy—NW; Trentino—Alto Adige, Northeast Italy—NE), (ii) the phytoplasma subtype (AT1–AO from NW; AT1 and AT2 from NE), and (iii) the psyllid haplotype (gt01, gt02, gt08–gt10, gt12–gt20). These models fitted using the R package lme4 (Bates et al. 2015) followed the initial structure:

$${\text{Phytoplasma}}\;{\text{Acquisition}}\, =\,{\text{Psyllid}}\;{\text{Origin}}\;{\text{Region}}\, +\,{\text{Phytoplasma}}\;{\text{Subtype}}\, +\,{\text{Psyllid}}\;{\text{Haplotype}}\, + \,\left({{1}|{\text{Cmel}}\;{\text{Lineage}}} \right)\, + \,\left({{1}|{\text{Tree}}} \right)\, + \,\varepsilon ,$$

where “Cmel Lineage” represents the newly emerged adults (full siblings) derived from the single mating couples, “Tree” represents the phytoplasma infected trees on which the psyllids were reared, and ε represents the residual unexplained error. All statistical analyses were done in R v.4.2.2 (R Core Team 2022), and models were repeated with exclusion of the outliers to test the robustness of the results. Details can be found in the SI.

## Results

### ‘*Candidatus *Phytoplasma mali’ infection rate in natural populations of *Cacopsylla melanoneura*

The phytoplasma natural infection rate was determined in the overwintered adults of *C. melanoneura* collected from apple orchards in Northwest (NW) and Northeast (NE) Italy. Among *C. melanoneura* from NW, three out of 43 tested individuals (6.98%) were infected with ‘*Ca.* P. mali’, whereas five out of 157 individuals (3.18%) were infected in the NE populations (Table 1). These five positive psyllids originated from three different populations collected in San Michele all’Adige (TN, 2 individuals), Bozen (BZ, 2 individuals) and Barbian (ET, 1 individual). The quantifiable samples showed an average concentration of around 1E + 06 copies of phytoplasma per insect in both the populations from NW and NE (Table 1).

**Table 1:** Natural infection rate of *Cacopsylla melanoneura* overwintered adults collected in Northern Italy

Sampling location	Insect population	Tested insects	Infected insects (nq)	Infection rate (%)		Phytoplasma copies per insect
*Valle d’Aosta (NW)*						
Aosta	AO1	22	2 (1)	9	6.98^a^	1.06E + 07
Saint-Pierre	AO2	21	1	5		7.90E + 06
*Trentino—Alto Adige (NE)*						
Brixen	BR	20	0	0	3.18^b^	–
Bozen	BZ	20	2 (1)	10		5.90E + 06
Barbian, Eisacktal	ET	20	1 (1)	5		nq
Laimburg	LB	20	0	0		–
Dorf Tirol, Meran	M	20	0	0		–
San Michele all’Adige	TN	20	2 (2)	10		nq
Schluderns, Vinschgau	V1	18	0	0		–
Malles, Vinschgau	V2	19	0	0		–

Sampling locations and the corresponding insect population codes for Valle d’Aosta (Northwest, NW) and Trentino—Alto Adige (Northeast, NE) are indicated in the first two columns. The geographic coordinates of the sampling locations are listed in Table S1. The phytoplasma concentration is expressed as phytoplasma copies per insect. The number of positive insects for which a precise quantification was not possible is indicated next to the total number of infected insects (nq, non-quantifiable).

^a^Mean infection rate for Valle d'Aosta (NW)

^b^Mean infection rate for Trentino - Alto Adige (NE)

### Experimental acquisition rate of ‘*Candidatus* Phytoplasma mali’ in newly emerged adults of *Cacopsylla melanoneura* derived from single mating couples

**Fig. 2:**
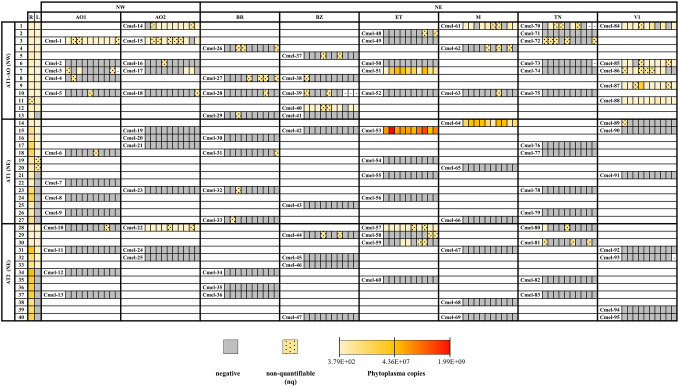
Heatmap of the acquisition experiment showing the ‘*Candidatus* Phytoplasma mali’ concentration in the newly emerged adults of *Cacopsylla melanoneura*. The trees (*n* = 40) were infected with ‘*Ca.* P. mali’ subtypes AT1–AO from the region of Valle d’Aosta (Northwest, NW), AT1 or AT2 from the region of Trentino—Alto Adige (Northeast, NE), Italy. The phytoplasma concentration of the roots (*R*) and leaves (*L*) of the infected trees are shown in the first two columns. A total of 942 individual psyllids from 95 lineages (Cmel1 to Cmel95) were analyzed: each square corresponds to one insect (around 10 insects per lineage; -, not analyzed). The *C. melanoneura* individuals belong to the following populations: AO1 and AO2 from NW; BR, BZ, ET, M, TN and V1 from NE. Sampling locations and the corresponding insect population codes are listed in Table 1. Heatmap legend: gray = no phytoplasma detected; yellow dotted = phytoplasma positive but non-quantifiable; from yellow to red = increasing copy number of phytoplasma. The quantification values of the *C. melanoneura* lineages are reported in Tables S2, S3 and S4

Even though the roots of all trees tested positive for phytoplasma at the start of the experiment (March), not all leaves had a detectable amount of phytoplasma when the newly emerged adults of *C. melanoneura* appeared (April) (Fig. 2, S1). Almost all trees infected with subtype AT1–AO had positive leaves (13/14 trees), whereas only about half of the trees infected with either AT1 or AT2 had a detectable amount of phytoplasma (7/14 and 6/13, respectively) (Fig. 2). Considerable differences were observed in the concentrations of the three phytoplasma subtypes in roots and leaves (Figure S1). Trees infected with subtype AT1–AO had a similar concentration in the roots (1.55E + 06 ± 3.88E + 05 phytoplasma copies per 100 mg roots) and in the leaves (7.75E + 05 ± 3.29E + 05 phytoplasma copies per 100 mg leaves) (Figure S1). In contrast, subtypes AT1 and AT2 had a higher concentration in the roots (1.27E + 07 ± 2.31E + 06 and 2.18E + 07 ± 4.41E + 06 phytoplasma copies per 100 mg roots) than in the leaves (5.83E + 04 ± 5.43E + 04 and 3.76E + 03 ± 1.75E + 03 phytoplasma copies per 100 mg leaves) (Figure S1).

To assess which factors drive the acquisition process in newly emerged adults of *C. melanoneura*, we established 24 cross combinations representing the region of origin of the psyllids and the phytoplasma subtype (Fig. 1b). In total, we analyzed 942 newly emerged adults of *C. melanoneura* from 95 lineages (Fig. 2; Tables S2, S3, S4). All analyzed psyllid populations could acquire ‘*Ca.* P. mali’ irrespective of their origin, but with different efficiencies depending on the phytoplasma subtype of the trees (Table 2, Fig. 2). Overall, ‘*Ca.* P. mali’ subtype AT1–AO was acquired by a higher number of *C. melanoneura* individuals from both NW and NE. A total of 37.37% *C. melanoneura* individuals from NW (AO1, AO2) acquired subtype AT1–AO, but only 1.25% and 15.71% of individuals acquired the subtypes AT1 and AT2, respectively (Table 2). Overall, 37.23% of the individuals from NE acquired subtype AT1–AO, whereas 12.00% and 10.50% of the individuals from NE acquired the subtypes AT1 and AT2, respectively (Table 2). When the newly emerged individuals developed on phytoplasma infected trees with negative leaves, they did not acquire ‘*Ca.* P. mali’, except for three individuals out of 942 (0.88%) from the lineages Cmel-29, Cmel-32, Cmel-33 (Fig. 2).

**Table 2:** Infection rate of *Cacopsylla melanoneura* newly emerged adults from the phytoplasma acquisition experiment

Phytoplasma subtype	Insect population		Tested insects	Infected insects (nq)	Infection rate (%)		Phytoplasma copies per insect ± SE
AT1–AO (NW)	NW	AO1	49	15 (7)	30.61	37.37^a^	1.02E+04 ± 5.91E+03
		AO2	50	22 (9)	44.00		3.28E+04 ± 1.08E+04
	NE	BR	40	9 (9)	22.50	37.23^a^	nq
		BZ	47	14 (7)	29.79		1.05E+04 ± 5.06E+03
		ET	50	12 (2)	24.00		7.95E+06 ± 3.22E+06
		M	30	12 (6)	40.00		4.80E+05 ± 2.86E+05
		TN	57	10 (8)	17.54		1.71E+03 ± 1.23E+03
		V1	50	45 (9)	90.00		1.73E+06 ± 8.20E+05
		Total	373	139 (57)	37.27		1.66E+06 ± 5.62E+05
AT1 (NE)	NW	AO1	40	1 (1)	2.50	1.25^a^	nq
		AO2	40	0	0.00		–
	NE	BR	40	3 (3)	7.50		nq
		BZ	20	0	0.00		–
		ET	40	10	25.00	12.00^a^	1.62E+08 ± 6.24E+07
		M	30	10	33.33		1.66E+07 ± 6.67E+06
		TN	40	0	0.00		–
		V1	30	1 (1)	3.33		nq
		Total	280	25 (5)	8.93		9.96E+07 ± 1.86E+07
AT2 (NE)	NW	AO1	40	1 (1)	2.50	15.71^a^	nq
		AO2	30	10 (2)	33.33		8.46E+04 ± 2.93E+04
	NE	BR	30	0	0.00	10.50^a^	–
		BZ	40	2 (2)	5.00		nq
		ET	40	16 (6)	40.00		1.59E+05 ± 5.82E+04
		M	30	0	0.00		–
		TN	40	5 (4)	16.67		7.82E+02 ± 7.71E+02
		V1	39	0	0.00		–
		Total	289	34 (15)	11.76		7.97E+04 ± 1.37E+04
Overall		Total	942	198 (77)	21.02		1.10E+07 ± 3.43E+06

Insects were reared on apple trees infected with ‘*Candidatus* Phytoplasma mali’ subtypes AT1–AO from the region of Valle d’Aosta (NW, Northwest), AT1 or AT2 from the region of Trentino—Alto Adige (NE, Northeast). Sampling locations and the corresponding insect population codes are listed in Table 1. The number of positive insects for which a precise quantification was not possible is indicated next to the total number of infected insects (nq, non-quantifiable). The phytoplasma concentration is expressed as phytoplasma copies per insect (± SE, standard error)

^a^Mean infection rate per region. The quantification values of the single *C. melanoneura* lineages are reported in Table S2, S3 and S4

While we observed differences between lineages from the same population reared on different trees and phytoplasma subtypes, the full siblings from a single mating couple had a similar infectious status, meaning that the newly emerged adults were either all positive or all negative and harbored a comparable phytoplasma concentration (Fig. 2). The acquisition efficiency varied also between lineages reared on the same tree: not all lineages could acquire the phytoplasma with the same rate. For instance, on tree number three, lineages Cmel-1 (AO1) and Cmel-15 (AO2) had a higher acquisition efficiency compared to lineages Cmel-49 (ET) and Cmel-72 (TN), which remained uninfected (Fig. 2). Among the selected populations, V1 (NE) had the highest acquisition rate: 90% of the newly emerged adults (5/5 lineages) acquired specifically subtype AT1–AO (NW). Likewise, the populations from AO1 and AO2 acquired only subtype AT1–AO, with the exception of Cmel-22 that could acquire subtype AT2 (Fig. 2). At least one lineage from the ET population acquired each of the three different subtypes. The M population had positive individuals for both AT1–AO and AT1 subtypes, whereas a few individuals of BZ acquired exclusively the subtype AT1–AO. Only a few individuals from BR and TN could acquire the phytoplasma subtypes AT1–AO and AT1 (Fig. 2, Table 2).

**Fig. 3:**
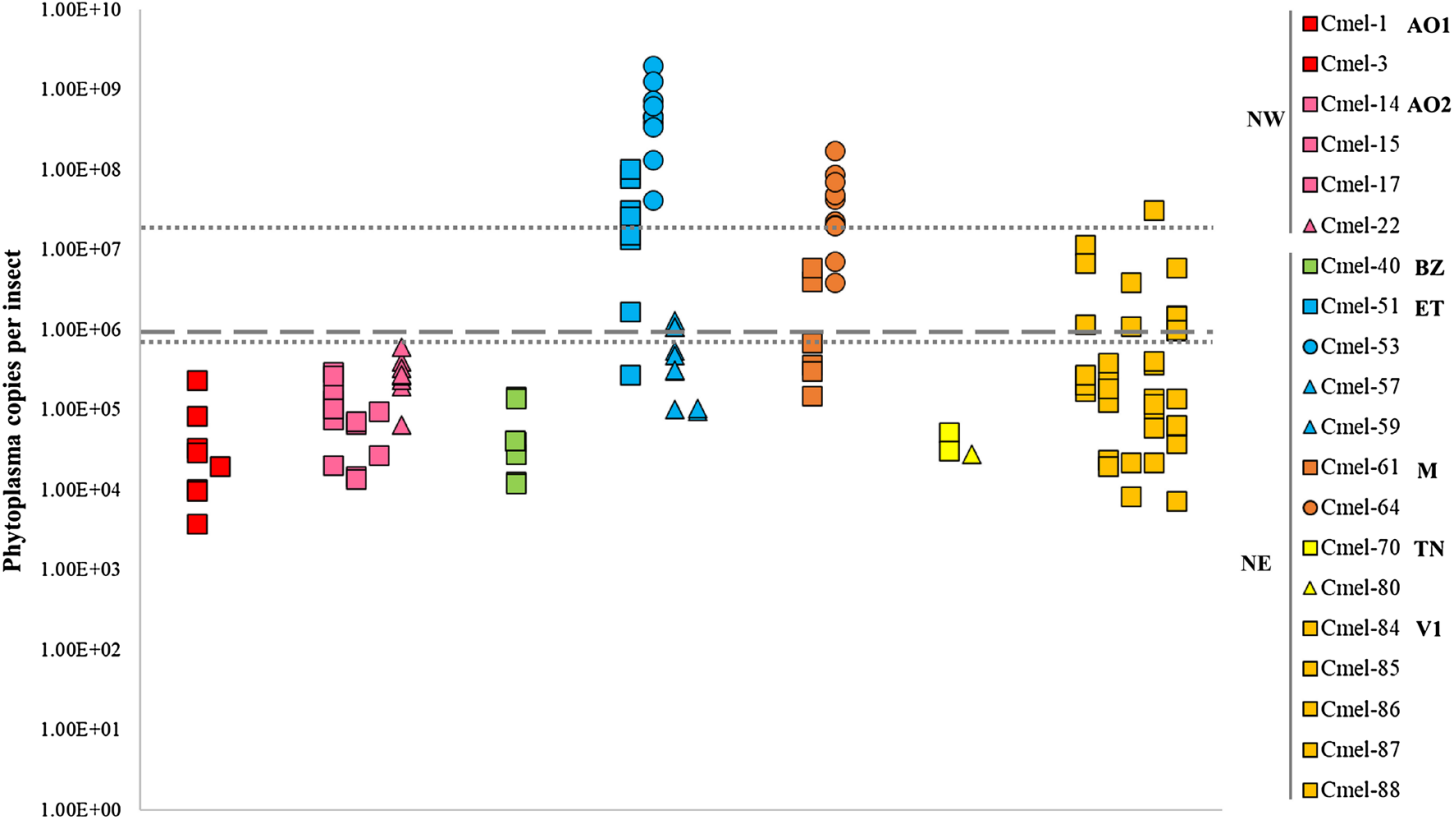
Quantification of ‘*Candidatus* Phytoplasma mali’ in newly emerged adults of *Cacopsylla melanoneura* from the phytoplasma acquisition experiment: results of positive, quantifiable single individuals are shown in Log10 scale. Insect populations from the region of Valle d’Aosta (Northwest, NW) in red (AO1) and pink (AO2); from the region of Trentino—Alto Adige (Northeast, NE) in yellow (TN), green (BZ), light blue (ET), orange (V1) and brown (M). Sampling locations and the corresponding insect population codes are listed in Table 1. Insects were reared on apple trees infected with ‘*Ca.* P. mali’ subtype AT1–AO (squares), AT1 (circles) or AT2 (triangles). The dashed line indicates the concentration required for phytoplasma transmission established for *C. picta* in Mayer et al., (2009). The dotted lines refer to the study of Candian et al. (2020): The upper dotted line represents the average phytoplasma concentration measured around 250 days after *C. melanoneura* migrated to conifers, while the lower dotted line shows the average phytoplasma concentration measured in newly emerged adults of *C. melanoneura* reared for 30 days on trees infected with AT1–AO. Both studies used the same quantification method (SYBR Green qPCR targeting the *rpl22* gene). The raw data from Candian et al., (2020) were used to calculate the phytoplasma concentration as “Phytoplasma copies per insect” to allow for comparison with the current study

The average concentration of ‘*Ca.* P mali’ was of 1.10E + 07 ± 3.43E + 06 copies per insect (Table 2). Cmel-53 (ET) reared on subtype AT1 harbored the highest concentration (6.47E + 08 ± 1.76E + 08 phytoplasma copies per insect; Fig. 2, Table S3), whereas Cmel-3 (AO1) and Cmel-80 (TN) were the lineages with the lowest concentration and were reared on trees infected with subtype AT1–AO and AT2, respectively (Fig. 2, Tables S2, S4). When comparing our results with previous studies that used the same quantification method, most of the tested newly emerged adults of *C. melanoneura* had a lower concentration than the phytoplasma transmission threshold determined by Mayer et al. (2009) and the concentration measured in newly emerged adults by Candian et al. (2020) (Fig. 3). Only a few individuals from ET, M and V1 populations harbored phytoplasma concentrations higher than the transmission threshold (Mayer et al. 2009) and similar to the ones measured in overwintering individuals around eight months after the migration to the conifers (Candian et al. 2020) (Fig. 3). For 77 out of 942 individuals (8.17%), we were not able to accurately quantify the phytoplasma concentration, and therefore, those individuals were defined as positive but non-quantifiable, nq (Fig. 2, Table 2).

### Haplotype diversity across the sampled populations of *Cacopsylla melanoneura*

**Fig. 4:**
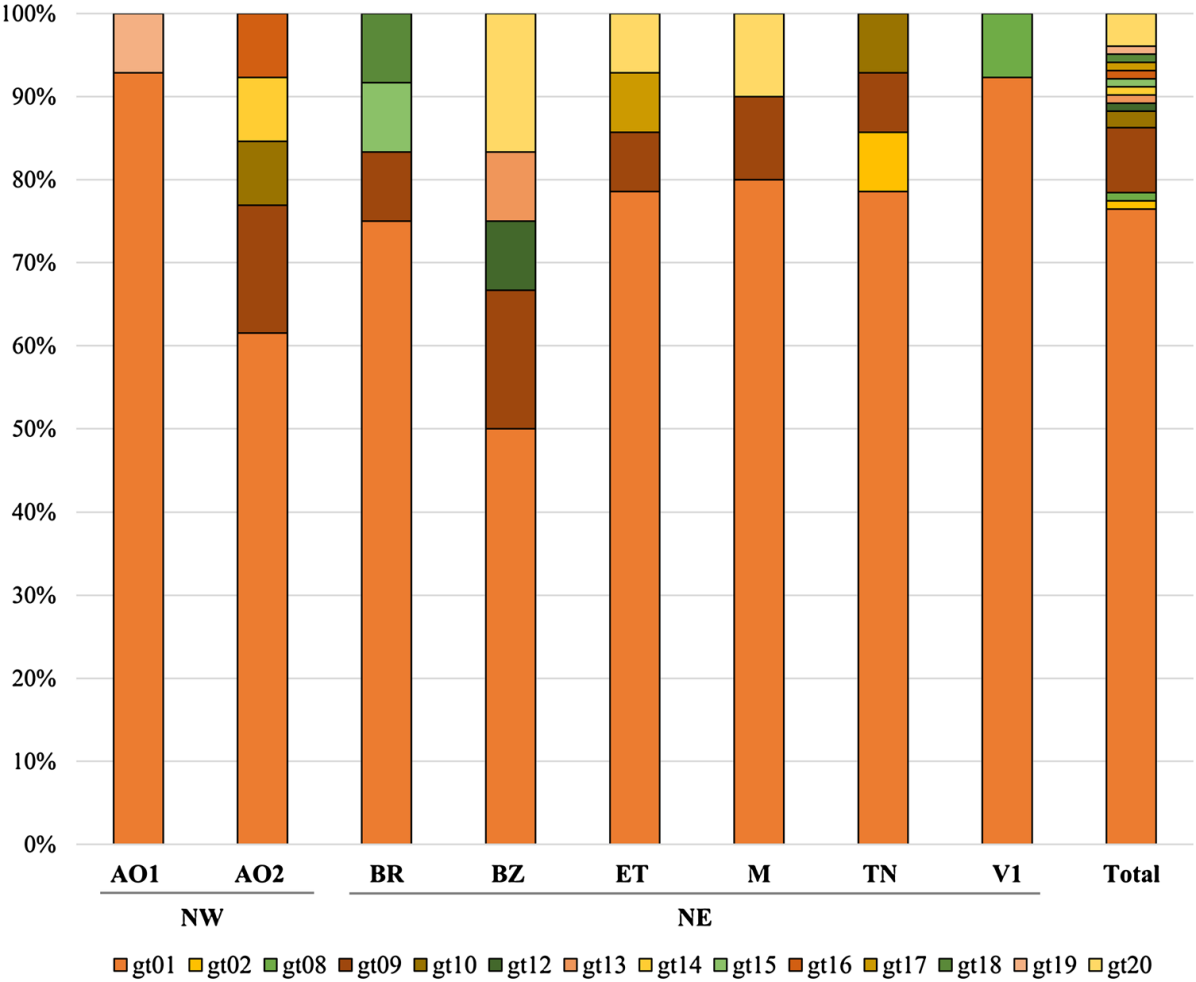
Haplotypes of the newly emerged adults of *Cacopsylla melanoneura* from the phytoplasma acquisition experiment based on the cytochrome oxidase subunit I (COI) gene. Insect populations from the region of Valle d’Aosta (Northwest, NW): AO1 (*n* = 14) and AO2 (*n* = 13); from the region of Trentino—Alto Adige (Northeast, NE): TN (*n* = 14), BR (*n* = 12), BZ (*n* = 12), ET (*n* = 14), V1 (*n* = 13) and M (*n* = 10). Sampling locations and the corresponding insect population codes are listed in Table 1

Sequencing of the COI mitochondrial gene of 190 individuals revealed a total of 14 haplotypes (Fig. 4, Table S5). Five haplotypes (gt01, gt02, gt08, gt09, gt10) were already described by Oettl and Schlink (2015), whereas 9 (gt12–gt20) are described here for the first time. They differ by one nucleotide from gt01 (gt12–gt18), gt07 (gt20) and gt09 (gt19) (Table S5). The populations from AO2 and BZ had the highest number of different haplotypes (5), followed by BR, ET, TN (4) and M (3), whereas in AO1 and V1 only two haplotypes were found (Fig. 4). Haplotype gt01 was the most common (77%) and was present in all populations. Other haplotypes were found with a lower frequency in both NW and NE, namely gt09 (7.84%), gt20 (3.92%) and gt10 (1.96%). In contrast, gt14, gt16 and gt19 (0.98% each) were only found in lineages from NW, while gt02, gt08, gt12, gt13, gt15, gt17 and gt18 (0.98% each) were identified only in NE lineages (Fig. 4, Tables S2–S4).

### Evaluation of the factors driving the acquisition process in newly emerged adults of *Cacopsylla melanoneura*

**Fig. 5:**
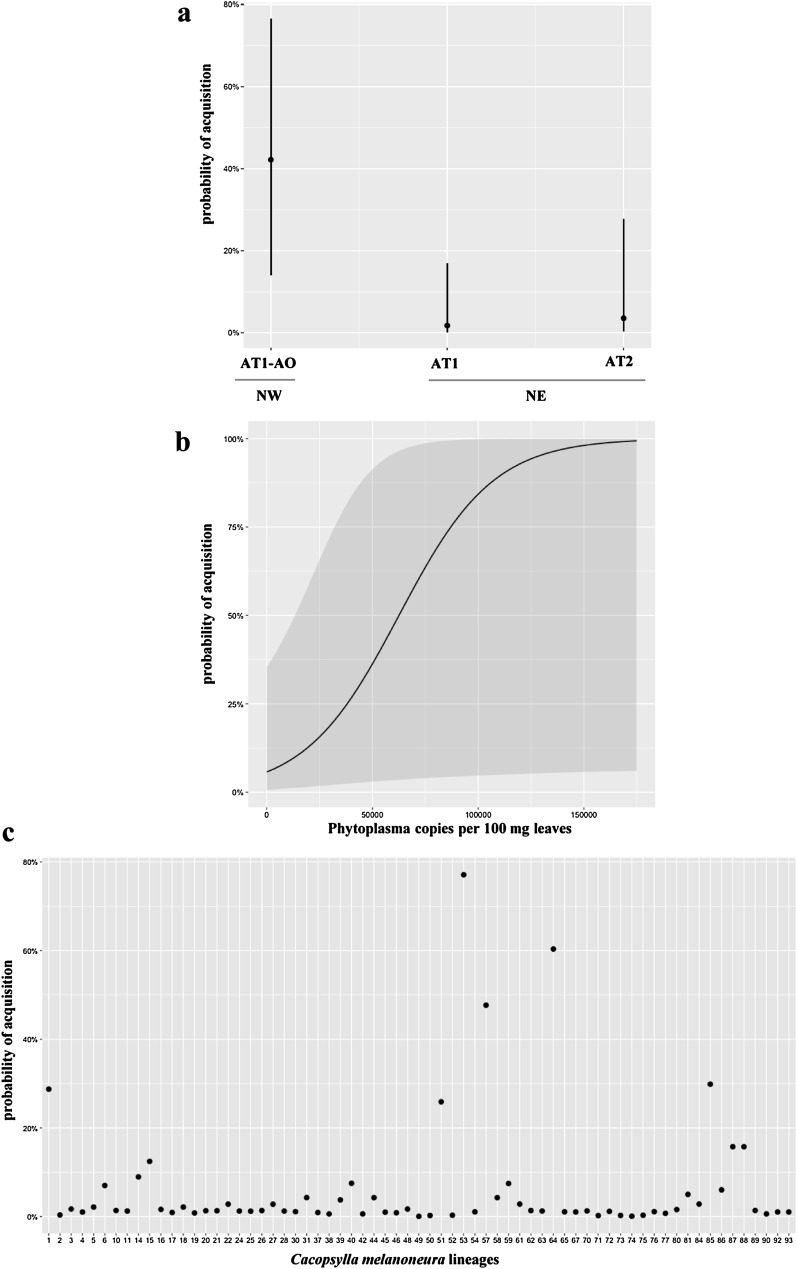
**a** Acquisition probability of the three ‘*Candidatus* Phytoplasma mali’ subtypes: AT1–AO from the region of Valle d’Aosta (Northwest Italy, NW) and AT1, AT2 from the region of Trentino—Alto Adige (Northeast Italy, NE). **b** Acquisition probability of ‘*Ca.* P. mali’ based on the phytoplasma concentration measured in the leaves of the infected trees used in the phytoplasma acquisition experiment. **c** Acquisition probability of ‘*Ca.* P. mali’ in the full siblings derived from the 95 *Cacopsylla* *melanoneura* single mating couples (lineages)

A positive association (Wald Test, *χ*^2^_1_ = 3.947, *P* = 0.047) was observed when examining the covariation between the presence of ‘*Ca.* P. mali’ in the newly emerged adults of *C. melanoneura* and the infected apple trees on which they had developed. The main factor driving the acquisition (yes/no) was the phytoplasma subtype, which explained 18.9% out of the total 19.2% of the variation in acquisition explained by the fixed effects of the model (Wald Test, *χ*^2^_2_ = 8.033, *P* = 0.018, Marginal *R*^2^_*GLMM*_ = 0.19). In particular, subtype AT1–AO was acquired by a greater number of individuals than AT1 (Tukey Contrast, *z* = 2.563, *P* = 0.028) and AT2 (Tukey Contrast, *z* = 2.115, *P* = 0.086). The predicted probability of acquisition (± 95% CI) was 42% (CI 14%, 77%) for AT1–AO, 2% (CI 0%, 17%) for AT1 and 0.04 (CI 0%, 28%) for AT2 (Fig. 5a). Both the region of origin (NW vs. NE) and the haplotype of the psyllid had no effect on the acquisition (Wald *χ*^2^_1_ = 0.565, *P* = 0.452 and Wald Test, *χ*^2^_11_ = 4.323, *P* = 0.959, respectively). When included in the model, the phytoplasma concentration in the leaves of the infected trees became the dominant effect, explaining 16.9% of the variation in the acquisition out of a total of 18.9% explained by the fixed effects of the model (Marginal *R*^2^_*GLMM*_ = 0.18) (Fig. 5b). While the phytoplasma subtype pattern remained qualitatively identical and was a required effect in the model, the redistribution of variance toward the phytoplasma concentration in the leaves reduced the significance of the phytoplasma subtype (Wald Test, *χ*^2^_2_ = 1.479, *P* = 0.477). The phytoplasma acquisition was also largely influenced by the psyllid lineage (Fig. 5c) in the initial model where it uniquely explained at least 17.5% of the total variance in acquisition (*χ*^2^_1_ = 118.4, *P* < 0.001) and after accounting for the variation in acquisition explained by the leaf concentration where it uniquely explained at least 20.9% of the total variance in acquisition (*χ*^2^_1_ = 119.6, *P* < 0.001).

Examining the acquisition of phytoplasma as a continuous variable for the subset of newly emerged adults with reliable qPCR data (i.e., excluding the non-quantifiable individuals, nq), a similar overall pattern was observed. The phytoplasma concentration in the leaves was again a prevalent factor and was positively associated with the presence of phytoplasma in the insect (Wald Test, *χ*^2^_1_ = 5.432, *P* = 0.019). However, there was also a clear effect of the region of origin (Wald Test, *χ*^2^_1_ = 155.5, *P* < 0.001), where newly emerged adults of *C. melanoneura* from NE acquired phytoplasma more successfully than those from NW. This model confirmed that subtype AT1–AO was the most frequently acquired (Wald Test, *χ*^2^_2_ = 19.28, *P* < 0.001). Additionally, we found a positive correlation between the phytoplasma concentration in the leaves and the region of origin of the insects (Wald Test, *χ*^2^_1_ = 7.712, *P* = 0.005). This interaction was particularly strong for the psyllids from NW, where the phytoplasma concentration in the leaves had a larger positive effect in these populations compared to the psyllids from NE, for which the phytoplasma concentration in the leaves had a lower importance. Again, the psyllid lineage was strongly associated with phytoplasma acquisition in the initial model (*χ*^2^_1_ = 3.13E + 08, *P* < 0.001) and after accounting for the leaves’ concentration (*χ*^2^_2_ = 3.12E + 08, *P* < 0.001).

When considering only *C. melanoneura* individuals with a positive and quantifiable amount of ‘*Ca.* P. mali’ (i.e., excluding the non-quantifiable, nq, and the negative individuals), we observed that several factors influence the concentration in the psyllids. Again, the phytoplasma subtype plays a central role explaining around 61% of the total 79% variation explained by fixed effects (Wald Test, *χ*^2^_2_ = 20.35, *P* < 0.001, Marginal *R*^2^_*GLMM*_ = 0.79). Subtype AT1 from NE was the most positively associated with a high concentration in the psyllids (Tukey Contrast: AT1 relative to AT1–AO, *z* = 1.120, *P* < 0.001; and relative to AT2, *z* = 1.356, *P* < 0.001), despite being acquired less often. There was, however, no effect of phytoplasma concentration in the leaves (Wald Test, *χ*^2^_1_ = 0.018, *P* = 0.893), explaining only 0.5% of the variation. The region of origin of the psyllid (Wald Test, *χ*^2^_1_ = 13.86, *P* < 0.001) explained around 21% of the variation. Interestingly, *C. melanoneura* from NE harbored higher phytoplasma concentrations than individuals from NW. Moreover, the psyllid lineage was significantly associated with the phytoplasma concentration in the psyllids in both the initial model (*χ*^2^_1_ = 4.34E + 05, *P* < 0.001) explaining at least 3.4% of the variance in concentration and after accounting for the variation in the concentration of the leaves (*χ*^2^_1_ = 4.34E + 05, *P* < 0.001), explaining at least 3.6% of variance in phytoplasma titer within the psyllids. There was also a marginal impact of the psyllid haplotype (Wald Test, *χ*^2^_3_ = 9.734, *P* = 0.021), explaining 8.3% of the variation in the concentration in the psyllids. However, we cannot pinpoint this effect to a specific haplotype since most of them occurred at low frequency in our *C. melanoneura* populations (Fig. 4).

## Discussion

Phytoplasmas are among the most economically relevant plant pathogens and are transmitted by various hemipteran insects (Rao et al. 2018; Bertaccini et al. 2019). Research on vector competence is crucial to understand the mechanisms and dynamics involved in their acquisition and transmission. In this work, we studied the dynamics of the pathosystem composed of the psyllid *C. melanoneura* and AP phytoplasma, ‘*Ca.* P. mali’. Previous studies focused on the natural infection rates and were based on experimental acquisition studies using a high number of randomly caught wild specimens feeding on plants infected with a single phytoplasma subtype (Mattedi et al. 2008; Oppedisano et al. 2019; Candian et al. 2020). Here, we compared the phytoplasma acquisition efficiency of a high number of newly emerged adults of *C. melanoneura* (full siblings) from populations with high (Northwest Italy, NW) and low (Northeast Italy, NE) vector efficiency. At the same time, we investigate the effect of the different ‘*Ca.* P. mali’ subtypes that are predominant in these areas. Our results highlight the factors influencing the acquisition efficiencies of phytoplasma and show the ability of *C. melanoneura* populations from both regions to acquire different phytoplasma subtypes.

The natural infection in the overwintered *C. melanoneura* was slightly higher in individuals from NW than from NE. However, the few individuals with a quantifiable amount of phytoplasma had on average similar concentrations. These results are in line with previous studies (Tedeschi et al. 2012; Oppedisano et al. 2019) and confirm once more that although *C. melanoneura* is not acknowledged as a vector in NE, overwintered individuals of natural population of *C. melanoneura* in both vector and non-vector regions harbored phytoplasma.

Data from the controlled acquisition experiment show that the infection rate in newly emerged adults of *C. melanoneura* developed on infected trees is driven mainly by the concentration and subtype of ‘*Ca.* P. mali’ in the leaves, where ‘*Ca.* P. mali’ AT1–AO from NW was the most frequently acquired subtype. The age of the infection might have played a role influencing the phytoplasma concentration in the leaves (all trees with AT1–AO and three trees with AT2 were grafted around 5 months before the experiment, whereas most of the plants with AT1 and AT2 from NE were prepared four years before). The newly grafted plants with AT1–AO might not have had enough time to adapt to the phytoplasma infection and to activate defense mechanisms that prevent its migration from the roots to the aerial parts of the tree (Musetti et al. 2010; Guerriero et al. 2012; Pagliari et al. 2017). However, since the three newly grafted AT2 plants had a similar concentration as the old ones, we assume that specific genetic features of AT1–AO from NW influenced the higher phytoplasma concentration in these plants, therefore favoring its acquisition by the psyllids. To further investigate the hypothesis that the phytoplasma subtype is a driving factor for the acquisition, whole-genome sequencing experiments are necessary to provide a more precise classification and a functional characterization of the genes and proteins that mediate its interactions with the plant and the insect. To date, little information is available about these ‘*Ca.* P. mali’ subtypes. AT1 is prevalent in NW and is mostly associated with *C. melanoneura*, whereas AT2 is the main subtype in NE and is predominantly associated with *C. picta* (Cainelli et al. 2004; Casati et al. 2010; Baric et al. 2011). The classification in these studies was based only on a few conserved genes (Jarausch et al. 2000; Martini et al. 2007), thus not providing any indication regarding the functional traits that might lead to a better colonization of the plant or a higher affinity to a specific species or population of *Cacopsylla* (i.e., *C. picta* vs. *C. melanoneura*, NW vs. NE populations). Our preliminary results from whole-genome sequencing of different regional ‘*Ca.* P. mali’ subtypes suggest that the AT1 subtypes from NW and NE have two distinct genomes (Calia et al., unpublished results), highlighting the need for a better taxonomic classification of AP phytoplasmas.

Surprisingly, the percentage of infected newly emerged individuals of *C. melanoneura* reared on AP phytoplasma infected trees were similar among populations from high (NW) and low (NE) vector efficiency regions. The infection rate of NW populations developed on trees infected with AT1–AO is slightly lower than the one measured in Candian et al. (2020). However, the phytoplasma concentration observed in our study was around 10-times lower, probably due to the fact that they allowed the newly emerged adults a longer acquisition period (10 days vs. 30 days). Our results further show that the phytoplasma concentration in the leaves had a higher positive influence on the acquisition in the populations from NW compared to NE. This highlights that individuals from NW are able to acquire phytoplasma at a lower concentration in the leaves. Thus, the role of newly emerged adults of *C. melanoneura* in low vector efficiency regions should not be underestimated. Young adults might still be present in apple orchards in late spring, when phytoplasma has fully migrated from the roots and colonized the tree aerial part (Pedrazzoli et al. 2008), thereby increasing their chance of feeding on an infected tree and acquiring phytoplasma. A clear example was the V1 population from NE that acquired the phytoplasma subtype AT1–AO from NW very efficiently. This population is from an area where the number of infected trees decreased significantly in the past years (Fischnaller et al. 2017); nevertheless, they were still able to acquire a phytoplasma subtype from a geographically distant region, where *C. melanoneura* is considered the only vector (Tedeschi et al. 2012).

Together with the phytoplasma subtype and its concentration in the leaves, the psyllid lineage is another key factor driving phytoplasma acquisition. In fact, while newly emerged siblings derived from a single mating couple of *C. melanoneura* had a similar infection status, we observed strong variation in the infectious status of psyllid lineages grown on the same tree (for example, tree 3 and 15). All analyzed individuals either did not or did acquire phytoplasma with a comparable concentration, apart from a few individuals (especially in the BR and TN populations from NE) where only one or two siblings belonging to different lineages got infected. Interestingly, when the newly emerged individuals of *C. melanoneura* developed on infected plants with negative leaves, meaning that the phytoplasma was still only located in the root system, only three out of the 942 analyzed psyllid individuals (0.3%) resulted positive. This suggests that either the phytoplasma was present in the aerial parts of the tree at a very low concentration (below our detection limit) or was very unevenly distributed in the canopy, thus escaped detection.

After successful acquisition, the ‘*Ca*. P. mali’ concentration in the newly emerged adults of *C. melanoneura* was influenced by several factors. The phytoplasma subtype played again a central role. However, subtype AT1 from NE could reach higher concentrations, even though it was the least frequently acquired subtype. This is clearly illustrated in the lineage Cmel-53 (ET) infected with subtype AT1 that harbored the highest concentration of ‘*Ca.* P. mali’. Even though AP outbreaks in the ET region were not as strong as in other areas of NE (Österreicher and Unterthurner 2014), AT1 is commonly found in this valley (Baric et al. 2011) and the possibility of *C. melanoneura* to carry such phytoplasma high concentrations should therefore not be underestimated. Our results also show that the phytoplasma concentration in the leaves has no effect on the phytoplasma concentration in the psyllids. Although the psyllid haplotype had a partial effect on the concentration in the insect, we could not identify a *C. melanoneura* haplotype that is related to a specific region or to a higher phytoplasma acquisition efficiency. Nevertheless, the region of origin and the psyllid lineage had a clear influence on the phytoplasma concentration measured in the psyllids. These results highlight that the genotype of the insect plays a role in the vector efficiency. The use of a single COI marker used in this study might therefore not be enough to detect small-scale genetic differences of the insects, and therefore, future studies are needed to perform a comparative genomics and gene expression analyses between vector and non-vector populations.

Although the current study focused specifically on the acquisition part of the AP transmission cycle, another key step is the phytoplasma replication inside the insect vector and its subsequent transmission. The pathogen multiplies inside the insect’s salivary glands, and it should reach a certain density before it can be effectively transmitted (Sugio et al. 2011). It was shown that phytoplasma can replicate and drastically increase its concentration inside *C. melanoneura* adults that had already migrated to conifers for the overwintering period, where the phytoplasma concentration reaches a plateau around five to eight months after migration (Candian et al. 2020). In this work, the phytoplasma concentrations in overwintered psyllids from the field and some of the newly emerged adults from the acquisition experiment were close to the threshold concentration for phytoplasma transmission established in *C. picta* by Mayer et al. (2009). For instance, newly emerged adults from the populations ET and M carried relatively high phytoplasma concentrations and therefore, might have been able to transmit phytoplasma to healthy plants. Previous studies showed that the concentration of phytoplasma in newly emerged psyllids reared on infected trees is higher compared to the concentration measured in overwintered individuals (Oppedisano et al. 2019; Candian et al. 2020). When comparing the transmission efficiency of phytoplasma in *C. melanoneura* to the other AP vector *C. picta* from NE, the concentration of the latter is around 10-times higher (Oppedisano et al. 2019), thus suggesting a different type of interaction between ‘*Ca.* P. mali’ and the two psyllid species from this region. The factors causing these discrepancies are currently not known. A recent study showed that even though no significant differences were found between infected and non-infected individuals of *C. melanoneura* and *C. picta* from NE, a higher number of low-abundance bacterial taxa were observed in non-infected *C. melanoneura* than in the infected individuals (Schuler et al. 2022). The opposite was true for the non-infected individuals of *C. picta*, which harbored a lower number of bacterial taxa compared to the infected individuals. Psyllids with a high phytoplasma concentration had more divergent microbiomes and, most importantly, there was a significant difference between the microbiomes of *C. melanoneura*, *C. picta* and eight non-AP transmitting *Cacopsylla* species, highlighting the potential role of the microbiome in the acquisition and transmission of phytoplasmas (Schuler et al. 2022). Therefore, further studies are needed to investigate the microbiome of *C. melanoneura* from NW and its potential interaction with ‘*Ca.* P. mali’.

In conclusion, we demonstrate that newly emerged adults of *C. melanoneura* from both high (NW) and low (NE) vector efficiency regions were able to acquire phytoplasma from infected trees in similar numbers and concentrations. The phytoplasma concentration in the leaves of the apple trees and the phytoplasma subtype drive the acquisition process in newly emerged adults of *C. melanoneura*, while the phytoplasma subtype and marginally the genetic features of the psyllid (region of origin, haplotype) affect how much phytoplasma is acquired and/or influence its replication kinetics within the insect body. It is important to keep in mind that the acquisition of phytoplasma does not necessarily lead to a successful transmission (Tedeschi and Alma 2004; Mayer et al. 2009; Oppedisano et al. 2019). Therefore, future studies are therefore needed to investigate whether these populations are capable to transmit AP phytoplasma.

## Author contributions

EC, MT, KJ and HS conceived and designed the research. EC, JD, VC, RT and HS performed the insect sampling. EC, MT, JD, CK, LŠS and HS conducted the experiment. EC, MT and CK conducted the molecular analysis. EC, MT and JMH analyzed the data. EC, LŠS and HS wrote the manuscript. All authors read and approved the manuscript.

### Supplementary Information

Below is the link to the electronic supplementary material.

Supplementary file1 (DOCX 69.1 KB)

Supplementary file2 (TIF 2228 KB)

Supplementary file3 (XLSX 11 KB)

Supplementary file4 (XLSX 13 KB)

Supplementary file5 (XLSX 13 KB)

Supplementary file6 (XLSX 13 KB)

Supplementary file7 (XLSX 11 KB)

## Data Availability

The sequences of the novel *C. melanoneura* haplotypes identified in this study were deposited in the NCBI GenBank database under the following accession numbers: OQ304120 to OQ304128.

## References

[CR1] Alma A, Lessio F, Nickel H (2019) Insects as phytoplasma vectors: ecological and epidemiological aspects. In: Bertaccini A, Weintraub PG, Rao GP, Mori N (eds) Phytoplasmas: plant pathogenic bacteria–II: transmission and management of phytoplasma–associated diseases. Springer, Singapore, pp 1–25. 10.1007/978-981-13-2832-9_1

[CR2] Altschul SF, Gish W, Miller W et al (1990) Basic local alignment search tool. J Mol Biol 215:403–410. 10.1016/S0022-2836(05)80360-2 2231712 10.1016/S0022-2836(05)80360-2

[CR3] Baric S, Berger J, Cainelli C et al (2011) Molecular typing of ‘*Candidatus* Phytoplasma mali’ and epidemic history tracing by a combined T-RFLP/VNTR analysis approach. Eur J Plant Pathol 131:573. 10.1007/s10658-011-9832-0 10.1007/s10658-011-9832-0

[CR4] Baric S, Dalla-Via J (2004) A new approach to apple proliferation detection: a highly sensitive real-time PCR assay. J Microbiol Methods 57:135–145. 10.1016/J.MIMET.2003.12.009 15003696 10.1016/J.MIMET.2003.12.009

[CR5] Bates D, Mächler M, Bolker B, Walker S (2015) Fitting linear mixed-effects models using lme4. J Stat Softw 67:1–48. 10.18637/jss.v067.i01 10.18637/jss.v067.i01

[CR6] Bertaccini A, Arocha-Rosete Y, Contaldo N et al (2022) Revision of the ‘*Candidatus* Phytoplasma’ species description guidelines. Int J Syst Evolut Microbiol 72:005353. 10.1099/ijsem.0.005353 10.1099/ijsem.0.005353 35471141

[CR7] Bertaccini A, Weintraub PG, Rao GP, Mori N (eds) (2019) Phytoplasmas: plant pathogenic bacteria—II: transmission and management of phytoplasma—associated diseases. Springer Singapore, Singapore

[CR8] Bosco D, D’Amelio R (2009) Transmission specificity and competition of multiple phytoplasmas in the insect vector. In: Weintraub G, Jones P (eds) Phytoplasmas-genomes, plant hosts and vectors. CABI, London, pp 233–249

[CR9] Brower AVZ, DeSalle R (1998) Patterns of mitochondrial versus nuclear DNA sequence divergence among nymphalid butterflies: the utility of wingless as a source of characters for phylogenetic inference. Insect Mol Biol 7:73–82. 10.1046/J.1365-2583.1998.71052.X 9459431 10.1046/J.1365-2583.1998.71052.X

[CR10] Cainelli C, Bisognin C, Vindimian ME, Grando MS (2004) Genetic variability of ap phytoplasmas detected in the apple growing area of Trentino (North Italy). Acta Hortic 657:425–430. 10.17660/ActaHortic.2004.657.68 10.17660/ActaHortic.2004.657.68

[CR11] Candian V, Monti M, Tedeschi R (2020) Temporal dynamics of ‘*Ca.* Phytoplasma mali’ load in the insect vector *Cacopsylla**melanoneura*. InSects 11(9):592. 10.3390/INSECTS11090592 32899174 10.3390/INSECTS11090592 PMC7563469

[CR12] Casati P, Quaglino F, Tedeschi R et al (2010) Identification and molecular characterization of ‘*Candidatus* Phytoplasma mali’ isolates in North-western Italy. J Phytopathol 158:81–87. 10.1111/J.1439-0434.2009.01581.X 10.1111/J.1439-0434.2009.01581.X

[CR13] Edgar RC (2004) Muscle: a multiple sequence alignment method with reduced time and space complexity. BMC Bioinform 5:1–19. 10.1186/1471-2105-5-113/FIGURES/16 10.1186/1471-2105-5-113/FIGURES/16 PMC517706 15318951

[CR14] Fiore N, Bertaccini A, Bianco PA et al (2018) Fruit Crop Phytoplasmas. In: Rao GP, Bertaccini A, Fiore N, Liefting LW (eds) Phytoplasmas: plant pathogenic bacteria–I: characterisation and epidemiology of phytoplasma–associated diseases. Springer, Singapore, pp 153–190

[CR15] Firrao G, Martini M, Ermacora P et al (2013) Genome wide sequence analysis grants unbiased definition of species boundaries in “*Candidatus* Phytoplasma”. Syst Appl Microbiol 36:539–548. 10.1016/J.SYAPM.2013.07.003 24034865 10.1016/J.SYAPM.2013.07.003

[CR16] Fischnaller S, Parth M, Messner M et al (2017) Occurrence of different *Cacopsylla* species in apple orchards in South Tyrol (Italy) and detection of apple proliferation phytoplasma in *Cacopsylla**melanoneura* and *Cacopsylla**picta*. Cicadina 17:37–51

[CR17] Frisinghelli C, Delaiti L, Grando MS et al (2000) *Cacopsylla**costalis* (Flor 1861), as a vector of apple proliferation in Trentino. J Phytopathol 148:425–431. 10.1046/J.1439-0434.2000.00403.X 10.1046/J.1439-0434.2000.00403.X

[CR18] Guerriero G, Giorno F, Ciccotti AM et al (2012) A gene expression analysis of cell wall biosynthetic genes in Malus x domestica infected by “*Candidatus* Phytoplasma mali”. Tree Physiol 32:1365–1377. 10.1093/TREEPHYS/TPS095 23086810 10.1093/TREEPHYS/TPS095 PMC4937989

[CR19] Hodkinson ID (2009) Life cycle variation and adaptation in jumping plant lice (Insecta: Hemiptera: Psylloidea): a global synthesis. J Nat Hist 43:65–179. 10.1080/00222930802354167 10.1080/00222930802354167

[CR20] Huang CT, Cho ST, Lin YC et al (2022) Comparative genome analysis of ‘*Candidatus* Phytoplasma luffae’ reveals the influential roles of potential mobile units in phytoplasma evolution. Front Microbiol 13:773608. 10.3389/fmicb.2022.773608 35300489 10.3389/fmicb.2022.773608 PMC8923039

[CR21] Janik K, Barthel D, Oppedisano T, Anfora G (2020) Apple proliferation a joint review. ISBN 9788878430549

[CR22] Janik K, Mithöfer A, Raffeiner M et al (2017) An effector of apple proliferation phytoplasma targets TCP transcription factors-a generalized virulence strategy of phytoplasma? Mol Plant Pathol 18:435–442. 10.1111/MPP.12409 27037957 10.1111/MPP.12409 PMC6638208

[CR23] Jarausch B, Tedeschi R, Sauvion N et al (2019) Psyllid Vectors. In: Bertaccini A, Weintraub PG, Rao GP, Mori N (eds) Phytoplasmas: plant pathogenic bacteria–II: transmission and management of phytoplasma–associated diseases. Springer, Singapore, pp 53–78

[CR24] Jarausch W, Saillard C, Helliot B et al (2000) Genetic variability of apple proliferation phytoplasmas as determined by PCR-RFLP and sequencing of a non-ribosomal fragment. Mol Cell Probes 14:17–24. 10.1006/mcpr.1999.0279 10722788 10.1006/mcpr.1999.0279

[CR25] Kube M, Mitrovic J, Duduk B et al (2011) Current view on phytoplasma genomes and encoded metabolism. Sci World J 2012:185942. 10.1100/2012/185942 10.1100/2012/185942 PMC3322544 22550465

[CR26] Kube M, Schneider B, Kuhl H et al (2008) The linear chromosome of the plant-pathogenic mycoplasma “*Candidatus* Phytoplasma mali”. BMC Genomics 9(1):1–14. 10.1186/1471-2164-9-306 18582369 10.1186/1471-2164-9-306 PMC2459194

[CR27] Luge T, Kube M, Freiwald A et al (2014) Transcriptomics assisted proteomic analysis of *Nicotiana occidentalis* infected by *Candidatus* Phytoplasma mali strain AT. Proteomics 14:1882–1889. 10.1002/pmic.201300551 24920314 10.1002/pmic.201300551

[CR28] Malagnini V, Pedrazzoli F, Gualandri V et al (2010) A study of the effects of ‘*Candidatus* Phytoplasma mali’ on the psyllid *Cacopsylla**melanoneura* (Hemiptera: Psyllidae). J Invertebr Pathol 103:65–67. 10.1016/J.JIP.2009.11.005 19932702 10.1016/J.JIP.2009.11.005

[CR29] Malagnini V, Pedrazzoli F, Papetti C et al (2013) Ecological and genetic differences between *Cacopsylla**melanoneura* (Hemiptera, Psyllidae) populations reveal species host plant preference. PLoS ONE 8:e69663. 10.1371/journal.pone.0069663 23874980 10.1371/journal.pone.0069663 PMC3712957

[CR30] Martini M, Ermacora P, Falginella L et al (2008) Molecular differentiation of “*Candidatus* Phytoplasma mali” and its spreading in Friuli Venezia Giulia region (North-East Italy). Acta Hort 781:395–402. 10.17660/ACTAHORTIC.2008.781.56 10.17660/ACTAHORTIC.2008.781.56

[CR31] Martini M, Lee I-M, Bottner KD et al (2007) Ribosomal protein gene-based phylogeny for finer differentiation and classification of phytoplasmas. Int J Syst Evol Microbiol 57:2037–2051. 10.1099/IJS.0.65013-0 17766869 10.1099/IJS.0.65013-0

[CR32] Mattedi L, Forno F, Cainelli C et al (2008) Research on *Candidatus* Phytoplasma mali transmission by insect vectors in Trentino. Acta Hortic 781:369–374. 10.17660/ActaHortic.2008.781.52 10.17660/ActaHortic.2008.781.52

[CR33] Mayer CJ, Jarausch B, Jarausch W et al (2009) *Cacopsylla**melanoneura* has no relevance as vector of apple proliferation in Germany. Phytopathology 99:729–738. 10.1094/PHYTO-99-6-0729 19453233 10.1094/PHYTO-99-6-0729

[CR34] Mayer CJ, Vilcinskas A, Gross J (2008a) Phytopathogen lures its insect vector by altering host plant odor. J Chem Ecol 34(8):1045–1049. 10.1007/S10886-008-9516-1 18600377 10.1007/S10886-008-9516-1

[CR35] Mayer CJ, Vilcinskas A, Gross J (2008b) Pathogen-induced release of plant allomone manipulates vector insect behavior. J Chem Ecol 34:1518–1522. 10.1007/s10886-008-9564-6 19031034 10.1007/s10886-008-9564-6

[CR36] Mayer CJ, Vilcinskas A, Gross J (2011) Chemically mediated multitrophic interactions in a plant–insect vector-phytoplasma system compared with a partially nonvector species. Agric for Entomol 13:25–35. 10.1111/j.1461-9563.2010.00495.x 10.1111/j.1461-9563.2010.00495.x

[CR37] Mittelberger C, Obkircher L, Oettl S et al (2017) The insect vector *Cacopsylla**picta* vertically transmits the bacterium ‘*Candidatus* Phytoplasma mali’ to its progeny. Plant Pathol 66:1015–1021. 10.1111/ppa.12653 10.1111/ppa.12653

[CR38] Monti M, Martini M, Tedeschi R (2013) EvaGreen real-time PCR protocol for specific “*Candidatus* Phytoplasma mali” detection and quantification in insects. Mol Cell Probes 27:129–136. 10.1016/j.mcp.2013.02.001 23474195 10.1016/j.mcp.2013.02.001

[CR39] Musetti R, Paolacci A, Ciaffi M et al (2010) Phloem cytochemical modification and gene expression following the recovery of apple plants from apple proliferation disease. Phytopathology 100:390–399. 10.1094/PHYTO-100-4-0390 20205543 10.1094/PHYTO-100-4-0390

[CR40] Namba S (2019) Molecular and biological properties of phytoplasmas. Proc Jpn Acad Ser B 95:401–418. 10.2183/pjab.95.028 31406061 10.2183/pjab.95.028 PMC6766451

[CR41] Oettl S, Schlink K (2015) Molecular identification of two vector species, *Cacopsylla**melanoneura* and *Cacopsylla**picta* (Hemiptera: Psyllidae), of apple proliferation disease and further common psyllids of Northern Italy. J Econ Entomol 108:2174–2183. 10.1093/jee/tov204 26453706 10.1093/jee/tov204

[CR42] Oppedisano T, Panassiti B, Pedrazzoli F et al (2019) Importance of psyllids’ life stage in the epidemiology of apple proliferation phytoplasma. J Pest Sci 93:49–61. 10.1007/S10340-019-01130-8 10.1007/S10340-019-01130-8

[CR43] Ossiannilsson F (1992) The Psylloidea (Homoptera) of Fennoscandia and Demark. Fauna Entomologica Scandinavica, E. J. Brill, Leiden, vol 26, 346 pp

[CR44] Österreicher J, Unterthurner M (2014) Starker Anstieg von Apfeltriebsucht im Burggrafenamt and Vinschgau. Obstbau Weinbau 51(2):52–54

[CR45] Pagliari L, Buoso S, Santi S et al (2017) Filamentous sieve element proteins are able to limit phloem mass flow, but not phytoplasma spread. J Exp Bot 68:3673. 10.1093/JXB/ERX199 28859375 10.1093/JXB/ERX199 PMC5853782

[CR46] Pedrazzoli F, Ciccotti AM, Bianchedi PL et al (2008) Seasonal colonisation behaviour of *Candidatus* Phytoplasma mali in apple trees in Trentino. Acta Hortic 781:483–488. 10.17660/ACTAHORTIC.2008.781.70 10.17660/ACTAHORTIC.2008.781.70

[CR47] R Core Team (2022) R: A language and environment for statistical computing. R Foundation for Statistical Computing, Vienna

[CR48] Rao GP, Bertaccini A, Fiore N, Liefting LW (2018) Phytoplasmas: plant pathogenic bacteria—I: Characterisation and epidemiology of phytoplasma—associated diseases. Springer, Singapore

[CR49] Schuler H, Dittmer J, Borruso L et al (2022) Investigating the microbial community of *Cacopsylla* spp. as potential factor in vector competence of phytoplasma. Environ Microbiol 24(10):4771–4786. 10.1111/1462-2920.16138 35876309 10.1111/1462-2920.16138 PMC9804460

[CR50] Seemüller E, Carraro L, Jarausch W, Schneider B (2011) Chapter 14: Apple proliferation phytoplasma. Virus Virus-like Dis Pome Stone Fruits. 10.1094/9780890545010.014 10.1094/9780890545010.014

[CR51] Seemüller E, Schneider B (2004) “*Candidatus* Phytoplasma mali”, “*Candidatus* Phytoplasma pyri” and *Candidatus* Phytoplasma prunorum’, the casual agents of apple proliferation, pear decline and European stone fruit yellows, respectively. Int J Syst Evol Microbiol 54:1217–1226. 10.1099/ijs.0.02823-0 15280295 10.1099/ijs.0.02823-0

[CR52] Sugio A, MacLean AM, Kingdom HN et al (2011) Diverse targets of phytoplasma effectors: from plant development to defense against insects. Annu Rev Phytopathol 49:175–195. 10.1146/annurev-phyto-072910-095323 21838574 10.1146/annurev-phyto-072910-095323

[CR53] Tedeschi R, Alma A (2004) Transmission of apple proliferation phytoplasma by *Cacopsylla**melanoneura* (Homoptera: Psyllidae). J Econ Entomol 97:8–13. 10.1093/JEE/97.1.8 14998121 10.1093/JEE/97.1.8

[CR54] Tedeschi R, Baldessari M, Mazzoni V et al (2012) Population dynamics of *Cacopsylla**melanoneura* (Hemiptera: Psyllidae) in Northeast Italy and its role in the apple proliferation epidemiology in apple orchards. J Econ Entomol 105:322–328. 10.1603/EC11237 22606799 10.1603/EC11237

[CR55] Tedeschi R, Nardi F (2010) DNA-based discrimination and frequency of phytoplasma infection in the two hawthorn-feeding species, *Cacopsylla**melanoneura* and *Cacopsylla**affinis*, in northwestern Italy. Bull Entomol Res 100:741–747. 10.1017/S0007485310000118 20569524 10.1017/S0007485310000118

[CR56] Weil T, Ometto L, Esteve-Codina A et al (2020) Linking omics and ecology to dissect interactions between the apple proliferation phytoplasma and its psyllid vector *Cacopsylla**melanoneura*. Insect Biochem Mol Biol 127:103474. 10.1016/j.ibmb.2020.103474 33007407 10.1016/j.ibmb.2020.103474

