# Supplementary material for: Multiple factors driving the acquisition efficiency of apple proliferation phytoplasma in Cacopsylla melanoneura
Source: J Pest Sci (2004). 2023 Oct 6;97(3):1299–314. doi: 10.1007/s10340-023-01699-1 (PMC11344730; doi:10.1007/s10340-023-01699-1)
Supplement: Supplementary file 1 — Supplementary file1 (DOCX 69.1 KB) [file 10340_2023_1699_MOESM1_ESM.docx]

**Supplementary material to**

**Multiple factors driving the acquisition efficiency of apple proliferation phytoplasma in *Cacopsylla melanoneura***

**Erika Corretto^1^, Massimiliano Trenti^2^, Liliya Štarhová Serbina^1^, James Malcolm Howie^3^, Jessica Dittmer^1,4^, Christine Kerschbamer^2^, Valentina Candian^5^, Rosemarie Tedeschi^5^, Katrin Janik^2,^ Hannes Schuler^1,6*^**

^1^Faculty of Agricultural, Environmental and Food Science, Free University of Bozen-Bolzano, Bozen-Bolzano, Italy

^2^Laimburg Research Centre, Pfatten-Vadena, Italy

^3^Department of Forest and Soil Sciences, University of Natural Resources and Life Sciences Vienna, BOKU, Vienna, Austria

^4^Current address: Université d’Angers, Institut Agro, INRAE, IRHS, SFR Quasav, Angers, France

^5^Department of Agricultural, Forest and Food Sciences (DISAFA), University of Turin, Grugliasco, Italy

^6^Competence Centre for Plant Health, Free University of Bozen-Bolzano, Italy

***Corresponding author:** [hannes.schuler@unibz.it](mailto:hannes.schuler@unibz.it)

**ORCID**

**Erika Corretto** 0000-0002-7767-5191

**Massimiliano Trenti** 0000-0002-9264-2179

**Liliya Štarhová Serbina** 0000-0002-5171-8253

**James Malcolm Howie** 0000-0001-7142-5100

**Jessica Dittmer** 0000-0002-2600-9201

**Christine Kerschbamer** 0000-0002-4295-4035

**Valentina Candian** 0000-0002-5460-4315

**Rosemarie Tedeschi** 0000-0003-4846-5045

**Katrin Janik** 0000-0003-0878-7402

**Hannes Schuler** 0000-0001-8307-9831

**PCR conditions**

**Plant material**

We performed a PCR analysis to characterize the subtype of ‘*Candidatus* Phytoplasma mali’ present in the infected material collected in the field and used to prepare the infected trees by grafting. Briefly, PCR amplification was performed in a total volume of 50 µL using 4 µL DNA, 10 µL 5X HF Buffer, 0.5 µL dNTP (10 mM each), 0.5 µL iProof Polymerase (Biorad) and 2.5 µL of each primer AP10 and AP13 (10 µM) (Jarausch et al. 2000) with the following thermal conditions: 30 sec at 98°C, 35 cycles of 10 sec at 98°C, 30 sec at 57°C, 1 min at 72°C and 5 min at 72°C.

**DNA extraction and quantification of phytoplasma in roots and leaves**

‘*Candidatus* Phytoplasma mali’ was detected with duplex TaqMan qPCR targeting the phytoplasma 16S rRNA gene and the chloroplast gene for the leucine tRNA as internal positive control as described in Baric and Dalla-Via (2004). Each sample was tested in triplicates in a total volume of 10 µL containing 5 µL of iQ Multiplex Powermix (Biorad), 0.9 µL of primers qAP16SF - qAP16SR (10 µM), 0.2 µL of primers cpLeuF – cpLeuR (10 µM), 0.02 µL of each TaqMan probe and 2 µL DNA. Thermal conditions were as follows: 95 °C for 3 min, 35 cycles of 95°C for 15 sec and 60°C for 1 min.

**DNA extraction and quantification of phytoplasma in insects**

First, we performed a real time PCR using primers targeting the insect single copy gene wingless (*wg*), qPSY-WG-F and qPSY-WG-R, to verify the quality of the extracted DNA (Brower and DeSalle 1998). Subsequently, the concentration of ‘*Candidatus* Phytoplasma mali’ was measured via SYBR qPCR with the primers targeting the *rpl22* gene, rpAP15f-mod and rpAP15r3 (Monti et al. 2013). The same qPCR conditions were used for both primer pairs. A total of 2 µL genomic DNA were mixed with 5 µL SYBR FAST qPCR Kit Master Mix (KapaBiosystems), 0.25 µL of 10 µM primers and 2.5 µL nuclease-free water. The thermal protocol consisted in an initial denaturation at 95°C for 20 sec, 35 cycles of 95°C for 3 sec and 60°C for 30 sec, followed by a melting curve ramp from 60 to 95°C with increments of 0.5°C every 5 sec (CFX384Touch Real-Time PCR Detection System; Bio-Rad).

**Genotyping of the *Cacopsylla melanoneura* families from the acquisition experiment**

PCR amplification was performed in a total volume of 25 µL using 2 µL DNA, 12.5 µL DreamTaq Hot Start PCR mix (Thermo Fisher), 1.75 µL of each primer VPm_COI_F2 and VPm_COI_R4 (10 µM), and 7 µL nuclease-free water (Oettl and Schlink 2015). The following thermal conditions were used: 3 min at 95°C, 40 cycles of 30 sec at 95°C, 30 sec at 46°C, 1 min at 72°C and finally 10 min at 72°C.

**Statistical analysis of the factors influencing phytoplasma acquisition**

We fitted a series of Generalized Linear Mixed Effects Models (GLME) to our acquisition data to test which factors are driving the acquisition of ‘*Candidatus* Phytoplasma mali’ in newly emerged *Cacopsylla melanoneura* from the acquisition experiment. The considered factors were (i) the psyllid’s region of origin (Valle d’Aosta, Northwest Italy- NW; Trentino – Alto Adige, Northeast Italy- NE), (ii) the phytoplasma subtype (AT1- AO from NW; AT1 and AT2 from NE) and (iii) the psyllid haplotype (gt01, gt02, gt08 to gt10, gt12 to gt20). These models, fitted using the R package lme4 (Bates et al. 2015) followed the initial structure:

Phytoplasma Acquisition =

Psyllid Origin Region + Phytoplasma Subtype + Psyllid Haplotype + (1|Cmel Family) + (1|Tree) + ε,

where “Cmel Family” represents the newly emerged adults (full siblings) derived from the single mating couples, “Tree” represents the phytoplasma infected trees on which the psyllids were reared, and ε represents the residual unexplained error.

All statistical analyses were done in R v.4.2.2 (R Core Team 2022), and models were repeated with exclusion of the outliers to test the robustness of the resultsThe model was run in three forms: first, to directly examine success or failure in acquisition the results of the phytoplasma acquisition experiment were coded as a binary variable (phytoplasma acquisition vs non-acquisition) and a GLME was fitted using a binomial error structure and logit link function. Second, for the subset of individuals for which an accurate phytoplasma quantification with qPCR was possible, the model was run to examine variation in phytoplasma acquisition as a continuous count concentration, using a Poisson error structure and log link function. Third, to examine the impact of each factor on the variation of the phytoplasma concentration in the psyllids, another Poisson model was fitted to the subset of individuals with positive (non-zero) concentrations. Finally, because the random effect of Tree explained a significant portion of variation, a further set of GLMEs were fitted including the concentration of phytoplasma in apple tree leaves as a covariate to examine the possibility that the concentration in the leaves impacted the phytoplasma acquisition (SI_DataAnalysisScript). In each case, individual fixed effects were tested using type-II Anova in the R package Car (Weisberg, Sanford 2019), after which two- and three-way interactions were explored (using Anova type = III), building upwards and downwards in fixed effect interaction complexity using AIC and Anova contrasts (Jones et al. 2022) to determine the optimal model form (SI_DataAnalysisScript). Model contrasts to test the impact of psyllid family were also conducted for each of the three model classes.

**References**

Baric S, Dalla-Via J (2004) A new approach to apple proliferation detection: a highly sensitive real-time PCR assay. Journal of Microbiological Methods 57:135–145. https://doi.org/10.1016/J.MIMET.2003.12.009

Bates D, Mächler M, Bolker B, Walker S (2015) Fitting Linear Mixed-Effects Models Using lme4. Journal of Statistical Software 67:1–48. https://doi.org/10.18637/jss.v067.i01

Brower AVZ, DeSalle R (1998) Patterns of mitochondrial versus nuclear DNA sequence divergence among nymphalid butterflies: the utility of wingless as a source of characters for phylogenetic inference. Insect Molecular Biology 7:73–82. https://doi.org/10.1046/J.1365-2583.1998.71052.X

Jarausch W, Saillard C, Helliot B, et al (2000) Genetic variability of apple proliferation phytoplasmas as determined by PCR-RFLP and sequencing of a non-ribosomal fragment. Molecular and Cellular Probes 14:17–24. https://doi.org/10.1006/mcpr.1999.0279

Jones E, Harden S, Crawley MJ (2022) The R Book, 3rd Edition. Wiley

Monti M, Martini M, Tedeschi R (2013) EvaGreen Real-time PCR protocol for specific “Candidatus Phytoplasma mali” detection and quantification in insects. Molecular and Cellular Probes 27:129–136. https://doi.org/10.1016/j.mcp.2013.02.001

Oettl S, Schlink K (2015) Molecular identification of two vector species, cacopsylla melanoneura and cacopsylla picta (Hemiptera: Psyllidae), of apple proliferation disease and further common psyllids of Northern Italy. Journal of Economic Entomology 108:2174–2183. https://doi.org/10.1093/jee/tov204

R Core Team (2022) R: A language and environment for statistical computing. R Foundation for Statistical Computing, Vienna, Austria

Weisberg, Sanford F John (2019) An R Companion to Applied Regression, Third. Sage, Thousand Oaks CA

**Figure legends**

**Figure S1:** Concentration of ‘*Candidatus* Phytoplasma mali’ in the roots **(a)** and leaves **(b)** of infected apple trees used in the phytoplasma acquisition experiment. The trees were prepared by grafting using plant material infected with different phytoplasma subtypes: AT1-AO from the region of Valle d’Aosta (Northwest Italy, NW) and AT1, AT2 from the region of Trentino – Alto Adige (Northeast Italy, NE). The leaves were collected at the same time as the newly emerged adults of *Cacopsylla melanoneura*. The average phytoplasma concentration expressed as phytoplasma copies per 100 mg plant material (± SE, standard error) is indicated above the bars; different letters indicate statistically different concentrations (T-test: *p* < 0.05).

**Tables**

**Table S1:** Geographical coordinates of all sampling locations of *Cacopsylla melanoneura* in Northern Italy (NW, Northwest; NE, Northeast).

| **Region** | **Insect Population** | **Location** | **Geographical coordinates** |
| --- | --- | --- | --- |
| **Valle d’Aosta**  **(NW)** | **AO1** | Aosta, Valle d’Aosta | 45°44'59.8"N 7°18'59.2"E |
|  | **AO2** | Saint-Pierre, Valle d’Aosta | 45°42'38.4"N 7°13'03.3"E |
| **Trentino - Alto Adige**  **(NE)** | **BR** | Brixen, Alto Adige | 46°43'24.9"N 11°39'45.2"E |
|  | **BZ** | Bozen, Alto Adige | 46°31'33.0"N 11°21'20.1"E |
|  | **ET** | Barbian, Eisacktal, Alto Adige | 46°35'15.7"N 11°30'44.2"E |
|  | **M** | Dorf Tirol, Meran, Alto Adige | 46°41'38.9"N 11°09'36.5"E |
|  | **TN** | San Michele all’Adige, Trentino | 46°11'25.0"N 11°08'06.5"E |
|  | **V1** | Schluderns, Vinschgau, Alto Adige | 46°40'25.0"N 10°34'18.3"E |
|  | **V2** | Malles, Vinschgau, Alto Adige | 46°41'25.3"N 10°32'01.7"E |

**Table S2:** Infection rate of *Cacopsylla melanoneura* newly emerged adults derived from the single mating couples (families) reared on apple trees infected with ‘*Candidatus* Phytoplasma mali’ subtype AT1-AO from the region of Valle d’Aosta (Northwest Italy, NW). We report the phytoplasma concentration expressed as phytoplasma copies per 100 mg of plant material. The concentration was measured in the roots, before starting the rearing, and in the leaves, when collecting the newly emerged adults. Sampling locations and the corresponding insect population codes are listed in Table 1. The insect haplotype has been assigned based on the cytochrome oxidase subunit I (COI) gene: gt01 (KM206163.1), gt02 (KM206165.1), gt08 (KM206172.1), gt09 (KM206167.1), gt14 (OQ304122), gt15 (OQ304123), gt20 (OQ304128). The number of positive insects for which a precise quantification was not possible is indicated next to the total number of infected insects (nq, non-quantifiable). The phytoplasma concentration is expressed as phytoplasma copies per insect (± SE, standard error).

| **Insect Family** | **Tree** | **Phytoplasma Copies per 100 mg Roots** | **Phytoplasma Copies per 100 mg Leaves** | **Insect Population** | **Insect Haplotype** | **Infected Insects (nq)** | **Total Tested Insects** | **Infection Rate (%)** | **Phytoplasma Copies per Insect (± SE)** |
| --- | --- | --- | --- | --- | --- | --- | --- | --- | --- |
| Cmel-1 | 3 | 2.39E+06 | 1.13E+06 | AO1 | gt01 | 10 (3) | 10 | 100.00 | 5.84E+04 ± 2.92E+04 |
| Cmel-2 | 6 | 3.98E+06 | 1.36E+04 | AO1 | gt01 | 0 | 10 | 0 | 0 |
| Cmel-3 | 7 | 5.68E+02 | 4.19E+03 | AO1 | gt01 | 3 (2) | 9 | 33.33 | 2.86E+03 ± 2.65E+03 |
| Cmel-4 | 8 | 7.10E+05 | 3.85E+03 | AO1 | gt01 | 1 (1) | 10 | 10.00 | nq |
| Cmel-5 | 10 | 6.60E+03 | 7.55E+05 | AO1 | gt01 | 1 (1) | 10 | 10.00 | nq |
| Cmel-14 | 1 | 9.08E+05 | 6.35E+05 | AO2 | gt01 | 9 (2) | 10 | 90.00 | 1.32E+05 ± 3.57E+04 |
| Cmel-15 | 3 | 2.39E+06 | 1.13E+06 | AO2 | gt01 | 9 (5) | 10 | 90.00 | 3.35E+04 ± 1.34E+04 |
| Cmel-16 | 6 | 3.98E+06 | 1.36E+04 | AO2 | gt14 | 1 (1) | 10 | 10.00 | nq |
| Cmel-17 | 7 | 5.68E+02 | 4.19E+03 | AO2 | gt01 | 2 | 10 | 20.00 | 1.23E+04 ± 9.18E+03 |
| Cmel-18 | 10 | 6.60E+03 | 7.55E+05 | AO2 | gt01 | 1 (1) | 10 | 10.00 | nq |
| Cmel-26 | 4 | 5.83E+05 | 1.16E+03 | BR | gt01 | 3 (3) | 10 | 30.00 | nq |
| Cmel-27 | 8 | 7.10E+05 | 3.85E+03 | BR | gt01 | 4 (4) | 10 | 40.00 | nq |
| Cmel-28 | 10 | 6.60E+03 | 7.55E+05 | BR | gt15 | 1 (1) | 10 | 10.00 | nq |
| Cmel-29 | 13 | 7.48E+05 | 0 | BR | gt01 | 1 (1) | 10 | 10.00 | nq |
| Cmel-37 | 5 | 4.05E+06 | 1.64E+06 | BZ | gt01 | 2 (2) | 10 | 20.00 | nq |
| Cmel-38 | 8 | 7.10E+05 | 3.85E+03 | BZ | gt20 | 1 (1) | 10 | 10.00 | nq |
| Cmel-39 | 10 | 6.60E+03 | 7.55E+05 | BZ | gt20 | 2 (2) | 7 | 28.57 | nq |
| Cmel-40 | 12 | 3.03E+06 | 4.04E+02 | BZ | gt09 | 9 (2) | 10 | 90.00 | 5.25E+04 ± 1.91E+04 |
| Cmel-41 | 13 | 7.48E+05 | 0 | BZ | gt09 | 0 | 10 | 0 | 0 |
| Cmel-48 | 2 | 5.35E+05 | 1.07E+05 | ET | gt01 | 2 (2) | 10 | 20.00 | nq |
| Cmel-49 | 3 | 2.39E+06 | 1.13E+06 | ET | gt09 | 0 | 10 | 0 | 0 |
| Cmel-50 | 6 | 3.98E+06 | 1.36E+04 | ET | gt01 | 0 | 10 | 0 | 0 |
| Cmel-51 | 7 | 5.68E+02 | 4.19E+03 | ET | gt01 | 10 | 10 | 100.00 | 3.82E+07 ± 1.11E+07 |
| Cmel-52 | 10 | 6.60E+03 | 7.55E+05 | ET | gt01 | 0 | 10 | 0 | 0 |
| Cmel-61 | 1 | 9.08E+05 | 6.35E+05 | M | gt01 | 8 (2) | 10 | 80.00 | 1.44E+06 ± 7.52E+05 |
| Cmel-62 | 4 | 5.83E+05 | 1.16E+03 | M | gt01 | 3 (3) | 10 | 30.00 | nq |
| Cmel-63 | 10 | 6.60E+03 | 7.55E+05 | M | gt20 | 1 (1) | 10 | 10.00 | nq |
| Cmel-70 | 1 | 9.08E+05 | 6.35E+05 | TN | gt01 | 5 (3) | 8 | 62.50 | 1.68E+04 ± 9.70E+03 |
| Cmel-71 | 2 | 5.35E+05 | 1.07E+05 | TN | gt01 | 0 | 10 | 0 | 0 |
| Cmel-72 | 3 | 2.39E+06 | 1.13E+06 | TN | gt01 | 5 (5) | 10 | 50.00 | nq |
| Cmel-73 | 6 | 3.98E+06 | 1.36E+04 | TN | gt02 | 0 | 9 | 0 | 0 |
| Cmel-74 | 7 | 5.68E+02 | 4.19E+03 | TN | gt01 | 0 | 10 | 0 | 0 |
| Cmel-75 | 10 | 6.60E+03 | 7.55E+05 | TN | gt01 | 0 | 10 | 0 | 0 |
| Cmel-84 | 1 | 9.08E+05 | 6.35E+05 | V1 | gt01 | 8 (1) | 10 | 80.00 | 2.26E+06 ± 1.28E+06 |
| Cmel-85 | 6 | 3.98E+06 | 1.36E+04 | V1 | gt01 | 9 (2) | 10 | 90.00 | 1.43E+05 ± 4.29E+04 |
| Cmel-86 | 7 | 5.68E+02 | 4.19E+03 | V1 | gt08 | 8 (4) | 10 | 80.00 | 8.48E+05 ± 5.90E+05 |
| Cmel-87 | 9 | 1.71E+06 | 1.41E+06 | V1 | gt01 | 10 (2) | 10 | 100.00 | 4.13E+06 ± 3.70E+06 |
| Cmel-88 | 11 | Unquantifiable | 4.38E+06 | V1 | gt01 | 10 | 10 | 100.00 | 1.12E+06 ± 5.90E+05 |

**Table S3:** Infection rate of *Cacopsylla melanoneura* newly emerged adults derived from the single mating couples (families) reared on apple trees infected with ‘*Candidatus* Phytoplasma mali’ subtype AT1 from the region of Trentino - Alto Adige (Northeast Italy, NE). We report the phytoplasma concentration expressed as phytoplasma copies per 100 mg of plant material. The concentration was measured in the roots, before starting the rearing, and in the leaves, when collecting the newly emerged adults. Sampling locations and the corresponding insect population codes are listed in Table 1. The insect haplotype has been assigned based on the cytochrome oxidase subunit I (COI) gene: gt01 (KM206163.1), gt09 (KM206167.1), gt10 (KM206173.1), gt16 (OQ304124). The number of positive insects for which a precise quantification was not possible is indicated next to the total number of infected insects (nq, non-quantifiable). The phytoplasma concentration is expressed as phytoplasma copies per insect (± SE, standard error).

| **Insect Family** | **Tree** | **Phytoplasma Copies per 100 mg Roots** | **Phytoplasma Copies per 100 mg Leaves** | **Insect Population** | **Insect Haplotype** | **Infected Insects (nq)** | **Total Tested Insects** | **Infection Rate (%)** | **Phytoplasma Copies per Insect (± SE)** |
| --- | --- | --- | --- | --- | --- | --- | --- | --- | --- |
| Cmel-6 | 18 | 2.49E+07 | 1.81E+03 | AO1 | gt01 | 1 (1) | 10 | 10.00 | nq |
| Cmel-7 | 22 | 3.78E+06 | 0 | AO1 | gt01 | 0 | 10 | 0 | - |
| Cmel-8 | 24 | 2.58E+07 | 0 | AO1 | gt01 | 0 | 10 | 0 | - |
| Cmel-9 | 26 | 9.10E+06 | 0 | AO1 | gt01 | 0 | 10 | 0 | - |
| Cmel-19 | 15 | 1.37E+07 | 1.18E+03 | AO2 | gt09 | 0 | 10 | 0 | - |
| Cmel-20 | 16 | 9.70E+06 | 7.09E+03 | AO2 | gt16 | 0 | 10 | 0 | - |
| Cmel-21 | 17 | 2.50E+07 | 7.32E+03 | AO2 | gt01 | 0 | 10 | 0 | - |
| Cmel-23 | 23 | 2.00E+07 | 0 | AO2 | gt01 | 0 | 10 | 0 | - |
| Cmel-30 | 16 | 9.70E+06 | 7.09E+03 | BR | gt09 | 0 | 10 | 0 | - |
| Cmel-31 | 18 | 2.49E+07 | 1.81E+03 | BR | gt01 | 1 (1) | 10 | 10.00 | nq |
| Cmel-32 | 23 | 2.00E+07 | 0 | BR | gt01 | 1 (1) | 10 | 10.00 | nq |
| Cmel-33 | 27 | 1.57E+07 | 0 | BR | gt01 | 1 (1) | 10 | 10.00 | nq |
| Cmel-42 | 15 | 1.37E+07 | 1.18E+03 | BZ | gt01 | 0 | 10 | 0 | - |
| Cmel-43 | 25 | 4.73E+06 | 0 | BZ | gt01 | 0 | 10 | 0 | - |
| Cmel-53 | 15 | 1.37E+07 | 1.18E+03 | ET | gt01 | 10 | 10 | 100.00 | 1.29E+07 ± 3.51E+06 |
| Cmel-54 | 19 | 9.98E+06 | Unquantifiable | ET | gt01 | 0 | 10 | 0 | - |
| Cmel-55 | 21 | 3.93E+06 | 0 | ET | gt01 | 0 | 10 | 0 | - |
| Cmel-56 | 24 | 2.58E+07 | 0 | ET | gt01 | 0 | 10 | 0 | - |
| Cmel-64 | 14 | 3.53E+06 | 6.82E+05 | M | gt01 | 10 | 10 | 100.00 | 9.99E+05 ± 3.06E+05 |
| Cmel-65 | 20 | 7.55E+06 | Unquantifiable | M | gt09 | 0 | 10 | 0 | - |
| Cmel-66 | 27 | 1.57E+07 | 0 | M | gt01 | 0 | 10 | 0 | - |
| Cmel-76 | 17 | 2.50E+07 | 7.32E+03 | TN | gt10 | 0 | 10 | 0 | - |
| Cmel-77 | 18 | 2.49E+07 | 1.81E+03 | TN | gt01 | 0 | 10 | 0 | - |
| Cmel-78 | 23 | 2.00E+07 | 0 | TN | gt01 | 0 | 10 | 0 | - |
| Cmel-79 | 26 | 9.10E+06 | 0 | TN | gt01 | 0 | 10 | 0 | - |
| Cmel-89 | 14 | 3.53E+06 | 6.82E+05 | V1 | gt01 | 1 (1) | 10 | 10.00 | nq |
| Cmel-90 | 15 | 1.37E+07 | 1.18E+03 | V1 | gt01 | 0 | 10 | 0 | - |
| Cmel-91 | 21 | 3.93E+06 | 0 | V1 | gt01 | 0 | 10 | 0 | - |

**Table S4:** Infection rate of *Cacopsylla melanoneura* newly emerged adults derived from the single mating couples (families) reared on apple trees infected with ‘*Candidatus* Phytoplasma mali’ subtype AT2 from the region of Trentino - Alto Adige (Northeast Italy, NE,). We report the phytoplasma concentration expressed as phytoplasma copies per 100 mg of plant material. The concentration was measured in the roots, before starting the rearing, and in the leaves, when collecting the newly emerged adults. Sampling locations and the corresponding insect population codes are listed in Table 1. The insect haplotype has been assigned based on the cytochrome oxidase subunit I (COI) gene: gt01 (KM206163.1), gt09 (KM206167.1), gt10 (KM206173.1), gt12 (OQ304120), gt13 (OQ304121), gt17 (OQ304125), gt18 (OQ304126), gt19 (OQ304127), gt20 (OQ304128). The number of positive insects for which a precise quantification was not possible is indicated next to the total number of infected insects (nq, non-quantifiable). The phytoplasma concentration is expressed as phytoplasma copies per insect (± SE, standard error).

| **Insect Family** | **Tree** | **Phytoplasma Copies per 100 mg Roots** | **Phytoplasma Copies per 100 mg Leaves** | **Insect Population** | **Insect Haplotype** | **Infected Insects (nq)** | **Total Tested Insects** | **Infection Rate (%)** | **Phytoplasma Copies per Insect (± SE)** |
| --- | --- | --- | --- | --- | --- | --- | --- | --- | --- |
| Cmel-10 | 28 | 1.44E+04 | 1.87E+04 | AO1 | gt19 | 1 (1) | 10 | 10.00 | nq |
| Cmel-11 | 31 | 3.85E+07 | 3.79E+02 | AO1 | gt01 | 0 | 10 | 0 | nq |
| Cmel-12 | 34 | 5.60E+07 | 0 | AO1 | gt01 | 0 | 10 | 0 | - |
| Cmel-13 | 37 | 3.10E+07 | 0 | AO1 | gt01 | 0 | 10 | 0 | - |
| Cmel-22 | 28 | 1.44E+04 | 1.87E+04 | AO2 | gt01 | 10 (2) | 10 | 100.00 | 5.93E+03 ± 1.04E+03 |
| Cmel-24 | 31 | 3.85E+07 | 3.79E+02 | AO2 | gt01 | 0 | 10 | 0 | - |
| Cmel-25 | 32 | 1.70E+07 | 1.43E+04 | AO2 | gt10 | 0 | 10 | 0 | - |
| Cmel-34 | 34 | 5.60E+07 | 0 | BR | gt01 | 0 | 10 | 0 | - |
| Cmel-35 | 36 | 3.05E+07 | 0 | BR | gt18 | 0 | 10 | 0 | - |
| Cmel-36 | 37 | 3.10E+07 | 0 | BR | gt01 | 0 | 10 | 0 | - |
| Cmel-44 | 29 | 1.72E+03 | 3.24E+03 | BZ | gt01 | 2 (2) | 10 | 20.00 | nq |
| Cmel-45 | 32 | 1.70E+07 | 1.43E+04 | BZ | gt01 | 0 | 10 | 0 | - |
| Cmel-46 | 33 | 1.36E+07 | 7.02E+02 | BZ | gt12 | 0 | 10 | 0 | - |
| Cmel-47 | 40 | 2.63E+07 | 0 | BZ | gt13 | 0 | 10 | 0 | - |
| Cmel-57 | 28 | 1.44E+04 | 1.87E+04 | ET | gt01 | 10 (2) | 10 | 100.00 | 1.30E+04 ± 2.92E+03 |
| Cmel-58 | 29 | 1.72E+03 | 3.24E+03 | ET | gt20 | 2 (2) | 10 | 20.00 | nq |
| Cmel-59 | 30 | 6.70E+02 | 1.15E+04 | ET | gt17 | 4 (2) | 10 | 40.00 | 4.95E+02 ± 3.03E+02 |
| Cmel-60 | 35 | 3.20E+07 | 0 | ET | gt01 | 0 | 10 | 0 | - |
| Cmel-67 | 31 | 3.85E+07 | 3.79E+02 | M | gt01 | 0 | 10 | 0 | - |
| Cmel-68 | 38 | 1.57E+07 | 0 | M | gt01 | 0 | 10 | 0 | - |
| Cmel-69 | 40 | 2.63E+07 | 0 | M | gt01 | 0 | 10 | 0 | - |
| Cmel-80 | 28 | 1.44E+04 | 1.87E+04 | TN | gt09 | 2 (1) | 10 | 20.00 | 6.26E+01 ± 5.90E+01 |
| Cmel-81 | 30 | 6.70E+02 | 1.15E+04 | TN | gt01 | 3 (3) | 10 | 30.00 | - |
| Cmel-82 | 35 | 3.20E+07 | 0 | TN | gt01 | 0 | 10 | 0 | - |
| Cmel-83 | 37 | 3.10E+07 | 0 | TN | gt01 | 0 | 10 | 0 | - |
| Cmel-92 | 31 | 3.85E+07 | 3.79E+02 | V1 | gt01 | 0 | 10 | 0 | - |
| Cmel-93 | 32 | 1.70E+07 | 1.43E+04 | V1 | gt01 | 0 | 9 | 0 | - |
| Cmel-94 | 39 | 2.25E+07 | 0 | V1 | gt01 | 0 | 10 | 0 | - |
| Cmel-95 | 40 | 2.63E+07 | 0 | V1 | gt01 | 0 | 10 | 0 | - |

**Table S5**: Single nucleotide polymorphisms (SNP) found in the new *Cacopsylla melanoneura* genotypes based on the cytochrome oxidase subunit I (COI). The genotypes identified by Oettl and Schlink (2015) were used as references for the identification of SNPs after the alignment with MUSCLE (Edgar, 2004). Each SNP was confirmed in the chromatogram and was present in both individuals.

|  | **Position** | | **SNP** | |
| --- | --- | --- | --- | --- |
| **New haplotype (accession number)** | **New** | **Ref** | **New** | **Ref (gt01, KM206163.1)** |
| **gt12** (OQ304120**)** | 57 | 93 | C | T |
| **gt13** (OQ304121**)** | 277 | 313 | T | C |
| **gt14** (OQ304122**)** | 463 | 499 | G | A |
| **gt15** (OQ304123**)** | 502 | 538 | C | T |
| **gt16** (OQ304124**)** | 523 | 559 | G | A |
| **gt17** (OQ304125**)** | 529 | 565 | T | C |
| **gt18** (OQ304126**)** | 718 | 754 | G | A |
|  | **New** | **Ref** | **New** | **Ref (gt09, KM206167.1)** |
| **gt19** (OQ304127**)** | 474 | 514 | A | G |
|  | **New** | **Ref** | **New** | **Ref (gt07, KM206171.1)** |
| **gt20** (OQ304128**)** | 655 | 691 | A | G |
