# Supplementary figures and images for: Multiple factors driving the acquisition efficiency of apple proliferation phytoplasma in Cacopsylla melanoneura
Source: J Pest Sci (2004). 2023 Oct 6;97(3):1299–314. doi: 10.1007/s10340-023-01699-1 (PMC11344730; doi:10.1007/s10340-023-01699-1)

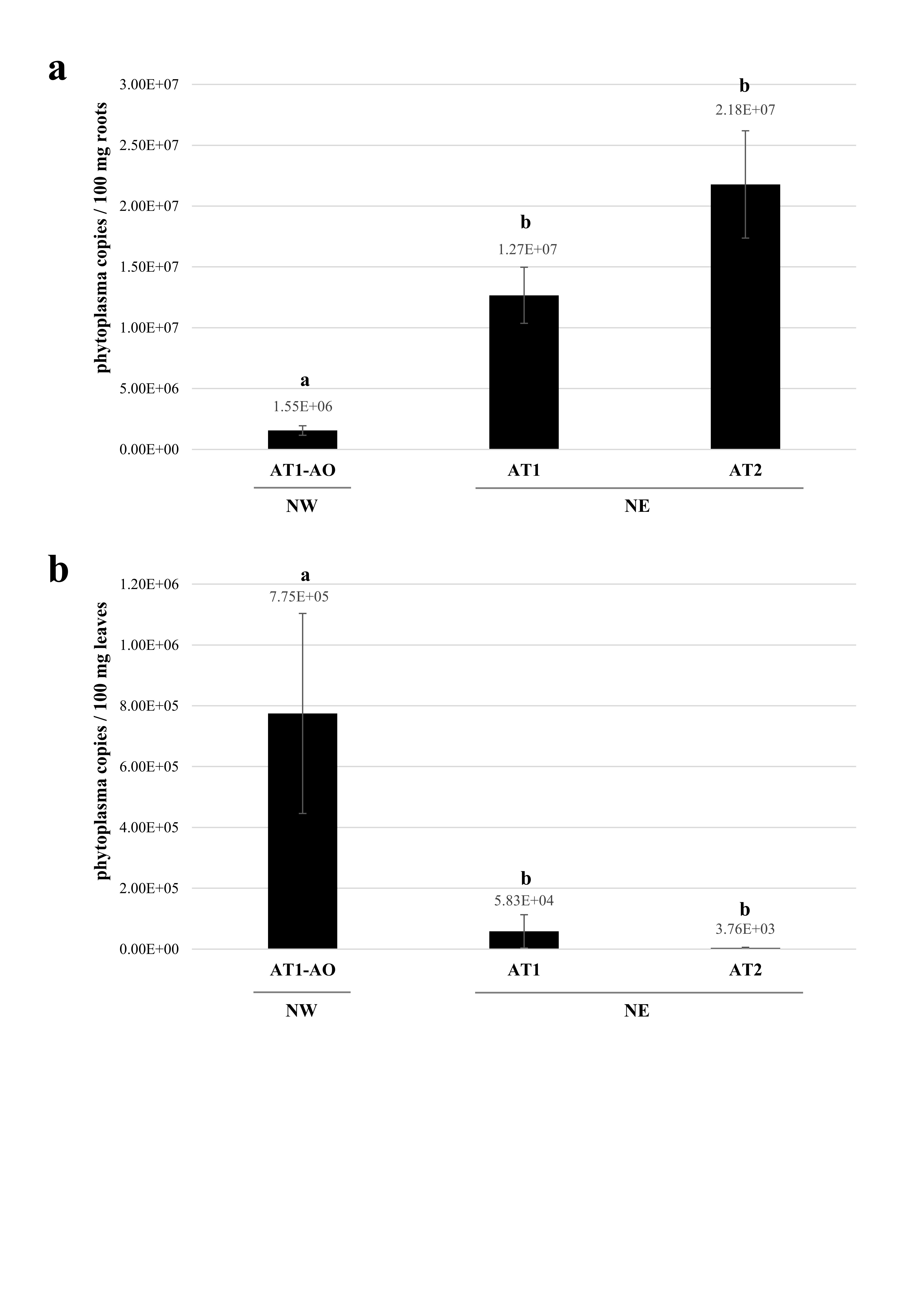

Supplement: Supplementary file 2 — Supplementary file2 (TIF 2228 KB) [file 10340_2023_1699_MOESM2_ESM.tif]
